# A comparative study of gut microbiota and metabolites in Tibetan sheep during cold and warm seasons

**DOI:** 10.3389/fvets.2026.1768985

**Published:** 2026-04-21

**Authors:** Qi-Tala An, Wenhao Li, Yaxiong Ren, Xia Liu, Liangwei Yao, Yuan Li, Xingxu Zhao, Yong Zhang, Peijian Feng, Xiaohua Du

**Affiliations:** 1 College of Veterinary Medicine, Gansu Agricultural University, Lanzhou, China; 2 Qilian Tibetan Sheep Industrial Development Research Institute of Qinghai Province, Qilian, China; 3 College of Animal Science and Veterinary Science, Qinghai University, Xining, China; 4 Research Center of High Altitude Medicine, Xining, China

**Keywords:** gut microbiota, microbial metabolites, microbiome research, season variation, Tibetan sheep

## Abstract

Tibetan sheep, a vital livestock species adapted to the extreme hypoxia, low temperatures, and intense radiation of the Qinghai–Tibet Plateau, rely on gastrointestinal microbiota for ecological balance and host nutrition, metabolism, and immunity. However, the possible associations of gut microbiota and metabolites with seasonal phenology remain unclear. Integrating biochemical, metagenomic, and metabolomic analyses, this study investigated seasonal variations in serum indices, microbial communities, and metabolites to inform enhanced breeding strategies. Analysis of forage nutritional composition showed that warm-season forages had significantly higher concentrations of dry matter (DM), crude protein (CP), and ether extract (EE) (*p* < 0.01), whereas cold-season forages were characterized by significantly greater levels of neutral detergent fiber (NDF) and acid detergent fiber (ADF) (*p* < 0.01). Correspondingly, serum analysis revealed significantly higher warm-season concentrations of alanine aminotransferase, total cholesterol, creatinine, and urea nitrogen compared with the cold season (*p* < 0.01). Gut microbiota composition shifted seasonally, with *Bacteroides* dominating in warm seasons and *Bacillus* predominating in cold seasons. Functional metagenomics indicated cold-season enrichment in pathways related to carbon metabolism, ABC transporters, aminoacyl-tRNA biosynthesis, pyruvate metabolism, DNA replication, and methane metabolism (*p* < 0.01). Metabolomics identified elevated warm-season microbial metabolites (His-Met, leucylleucine, luteolin 7-glucoside, ursolic acid; *p* < 0.05) and higher cold-season compounds (melatonin, glabrol, prostaglandin E2; *p* < 0.05), with KEGG enrichment linking these to steroid hormone biosynthesis, fatty acid metabolism, bile acid synthesis, and propanoate pathways. These findings suggest possible associations between seasonal extremes and: (1) modulation of nutrient metabolism (e.g., secondary bile acids and short-chain fatty acids); (2) activation of stress-response pathways (e.g., pentose phosphate pathway, ABC transporters, and DNA replication); and (3) immune regulation mediated by bioactive metabolites. Cold-season enrichment in DNA repair and energy-production pathways may be associated with responses to oxidative stress, whereas warm-season shifts in lipid metabolism are consistent with increased nutrient availability. Fluctuations in key metabolites—such as elevated melatonin in cold seasons and elevated ursolic acid in warm seasons—likely reflect adaptations related to thermoregulation and antioxidant defense. This work provides foundational insights into microbiota–host interactions under extreme environmental conditions, supporting the optimization of supplementation, probiotic use, and sustainable husbandry on the Qinghai–Tibet Plateau.

## 1 Introduction

Tibetan sheep represent a crucial genetic resource and economically vital livestock species uniquely adapted to the extreme hypoxic, high-altitude environment of the Qinghai-Tibet Plateau. Through long-term adaptive evolution, they have emerged as the dominant livestock breed in this oxygen-deprived region, with current herd sizes exceeding approximately 40 million head–constituting China’s largest sheep population (1). This breed exhibits exceptional adaptive traits including hypoxia tolerance, cold resistance, efficient roughage utilization, and disease resilience. The phenological shifts in alpine meadow forage between cold and warm seasons on the Qinghai-Tibet Plateau significantly influence the composition, structure, and functional dynamics of the Tibetan sheep gut microbiota and its metabolites (2). Critically, nutritional scarcity during cold seasons precipitates declines in production performance and economic value, substantially constraining the development of the Tibetan sheep industry.

Tibetan sheep occupy a central position in the pastoral economy and livelihood security of communities on the Qinghai–Tibet Plateau. Early studies on plateau grasslands and grazing systems have shown that productivity in alpine rangelands is strongly seasonal; traditional pastoralism relies heavily on forage supply during the summer–autumn growing season and mitigates forage scarcity in winter and spring through seasonal rotational grazing and transhumance (3). Recent studies on Tibetan sheep, together with official statistics, likewise indicate that this species has a large population size and provides substantial industrial support in major producing regions such as Qinghai. The phenology of alpine grasslands (green-up–peak growth–senescence) drives cyclical, year-round fluctuations in forage nutritional composition: forage in the warm season generally contains higher crude protein and energy, whereas cold-season forage is characterized by increased fiber proportions and reduced nutrient availability; under natural grazing conditions, feed intake also varies markedly by season (4). In combination with seasonal pasture use and transhumant grazing patterns, these ecological processes collectively alter the spectrum of substrates available to ruminants, thereby potentially reshaping the composition and functional capacity of the gut microbiota and further influencing host nutrient metabolism and health status (5).

A substantial body of evidence indicates that ruminant gut microbiota exhibit pronounced seasonal plasticity. Studies of high-altitude ruminants represented by yak have shown that seasonal shifts in diet composition can induce reproducible transitions in gut microbial community structure and “enterotypes/community types” between cold and warm seasons, accompanied by a reconfiguration of functional modules related to nitrogen and energy utilization, thereby helping the host maintain energy homeostasis under cold-season conditions of low nutrition and severe cold (6). Further metagenomic investigations have also suggested that, under plateau grazing systems, the rumen microbiome displays seasonal fluctuations in composition, function, and stability, and that different species (e.g., yak versus cattle) may adopt distinct microbiome-mediated adaptive strategies in extreme environments (7). In addition, cross-species studies have reported that, within the same plateau ecological niche, seasonal diet can exert a stronger influence than host species per se in shaping differences in the distal gut microbiota (8).

Compared with yak, research on the seasonality of the gut microbiota in Tibetan sheep remains relatively limited. Existing work has shown that cold-season nutritional stress in Tibetan sheep is accompanied by changes in ruminal volatile fatty acid (VFA) profiles and microbial community structure, and these shifts are correlated with the expression of host genes involved in nutrient transport and epithelial barrier function, suggesting that a “microbe–metabolite–host tissue” axis may constitute a key regulatory chain (9). Another study using untargeted metabolomics of rectal contents reported pronounced divergence in metabolite profiles between cold and warm seasons, to clarify the relationship between the present work and our previous publication, the earlier study primarily focused on seasonal differences in untargeted metabolomic profiles of rectal contents in Tibetan sheep. In the present study, although part of the metabolomics dataset originated from the same sample cohort, the analytical focus is different. Here, the metabolomics data were reanalyzed and interpreted together with serum biochemical indices and newly generated shotgun metagenomic data to support cross-omics integration. Accordingly, the metabolomics results presented here are not intended as a duplicate report of the previous metabolomics study, but as part of a newly structured multi-omics framework with a different analytical emphasis and figure presentation. Involving pathways such as fatty acid metabolism, secondary bile acid biosynthesis, and propionate metabolism, implying that microbial metabolites may contribute to energy supply as well as immuno-inflammatory regulation (4). Moreover, multi-tissue investigations across grassland phenological stages have suggested potential associations between the rumen microbiota and neurotransmitter/endocrine indices, supporting the notion that the microbiota–gut–brain axis may participate in plateau seasonal adaptation (10). To address this gap, the present study aimed to clarify the effects of seasonal variation on the composition and structure of the gut microbiota in Tibetan sheep, to characterize microbial diversity and functional adaptation across cold–warm seasonal transitions, and to further explore host–microbe interactions. We conducted seasonal serum biochemical profiling of Tibetan sheep, together with metagenomic sequencing and untargeted metabolomic analyses of rectal content samples. These data delineate seasonal differences in gut microbial composition and functional dynamics, help elucidate nutrition-related host–microbiota interactions under cold stress, and provide a foundation for optimizing Tibetan sheep management and cold-season supplementation strategies.

## 2 Materials and methods

### 2.1 Experimental materials

Twelve clinically healthy female Tibetan sheep aged 1–2 years, with similar body condition and originating from the same herd in Yeniugou Township, Qilian County, Qinghai Province, were included in this study. The experiment followed a parallel-group, cross-sectional design. Six animals were sampled in the warm season (August 2023; YF-1), and another six independent animals were sampled in the cold season (December 2023; YF-2). After ear-tagging and group allocation, all animals were maintained under the same traditional grazing system on alpine meadow pasture without artificial supplementation.

At slaughter, one serum sample and one rectal content sample were collected from each animal. Serum biochemistry and untargeted metabolomics were performed using samples from all 12 animals (*n* = 6 per season). Shotgun metagenomic sequencing was conducted on a subset of rectal content samples (*n* = 3 per season; total *n* = 6). Unless otherwise stated, the individual animal was treated as the statistical unit in all analyses.

### 2.2 Determination of nutritional components in forage

Twenty-four hours prior to formal sampling, 12 standardized quadrats (0.5 m × 0.5 m) were systematically established within the experimental area. The quadrats were arranged in a “W”-shaped spatial distribution pattern, with a fixed interval of 30 m between adjacent quadrat centers. After placement, all plant species within each quadrat were systematically identified and recorded. Aboveground vegetation was then completely harvested using the ground-level clipping method. Fresh samples were immediately sealed in kraft paper sampling bags, preserved under low-temperature conditions, and subsequently used for forage nutritional analysis. This sampling protocol followed established phytosociological survey standards to ensure representativeness, completeness, and analytical timeliness of the collected samples.

Forage samples were dried in an oven at 65 °C until constant weight to determine dry matter (DM) content. The dried material was then ground to pass through a 1 mm sieve, and the resulting powder was stored in sealed bags for subsequent nutrient analysis. Crude protein (CP) content was determined according to GB/T 6432–2018. Ether extract (EE) content was measured following GB/T 6433–2006. Neutral detergent fiber (NDF) and acid detergent fiber (ADF) contents were analyzed using the method described by Van Soest et al. (11).

### 2.3 Measurement of serum biochemical indicators

Serum biochemical indices were measured using the Kjeldahl method and micromethod. The analyzed parameters included total protein (TP), albumin (ALB), globulin (GLB), alanine aminotransferase (ALT), aspartate aminotransferase (AST), creatinine (CRE), blood urea nitrogen (BUN), total cholesterol (TC), and triglyceride (TG).

### 2.4 Macrogenomic sequencing

#### 2.4.1 Library construction and sequencing

Total DNA was extracted from rectal content samples using the HiPure Stool DNA Kit (D3141, Guangzhou Meiji Biotechnology Co., Ltd., China) according to the manufacturer’s instructions. DNA concentration and purity were assessed using a NanoDrop 2000 spectrophotometer (Thermo Fisher Scientific, USA), and DNA integrity was evaluated by agarose gel electrophoresis.

Shotgun metagenomic libraries were constructed using the NEBNext^®^ Ultra™ DNA Library Prep Kit for Illumina^®^ (NEB#E7370L, New England Biolabs, USA). Briefly, genomic DNA was fragmented using a double-stranded DNA fragmentation enzyme, followed by end repair, phosphorylation, and dA-tailing. Sequencing adapters were then ligated, and DNA fragments with target insert sizes of 300–400 bp were purified and size-selected using AMPure XP beads (Beckman Coulter, Brea, CA, USA). The libraries were enriched by 12 cycles of PCR and purified again with AMPure XP beads. Library quality was assessed using an Agilent 2,100 Bioanalyzer (Agilent Technologies, USA), quantified by real-time PCR (ABI StepOnePlus, Life Technologies, USA), pooled, and sequenced on the Illumina NovaSeq X Plus platform in paired-end 150 bp mode (PE150).

#### 2.4.2 Data processing

Raw paired-end reads generated on the Illumina platform were processed using fastp (v0.18.0) for quality control. Reads containing more than 10% ambiguous bases (N), reads with ≥50% low-quality bases under the Q20 threshold, and adapter-contaminated reads were removed to obtain high-quality clean reads. To reduce host contamination, clean reads were screened against the sheep reference genome and potential host-derived reads were discarded before downstream microbial analyses.

*De novo* assembly of clean reads was performed using MEGAHIT (v1.1.2) with multiple k-mer sizes. Assembly statistics were calculated at the contig level; therefore, the N50 values reported in this study refer to assembled contigs rather than sequencing reads. The resulting clean reads and assembly-derived sequences were used for subsequent taxonomic annotation, community structure analysis, and functional annotation.

#### 2.4.3 Analyze statistics

All data were statistically analyzed using the SAS 9.2 statistical software. One-way analysis of variance (ANOVA) was performed on the relative abundances of the Tibetan sheep gut microbiota at the phylum and genus levels. If the results showed significant differences, the Least Significant Difference (LSD) method was used for multiple comparison tests, and a *p*-value less than 0.05 was considered statistically significant. The Welch’s *t*-test was conducted using the Vegan package in R language. Additionally, the Bray–Curtis distance matrix between samples was calculated based on the abundances of genes, species, or functional genes to evaluate the inter-group difference characteristics of Alpha diversity and Beta diversity. For clarity, “*n*” refers to biological replicates (individual animals) unless otherwise stated. For untargeted metabolomics, each biological sample was analyzed in both positive and negative ion modes, whereas pooled QC samples were used only for instrument monitoring and were not treated as biological replicates.

### 2.5 Non-targeted metabolomics sequencing

#### 2.5.1 LC–MS analysis sample preparation

Rectal fecal samples were slowly thawed at 4 °C. An appropriate amount of each sample was mixed with precooled methanol/acetonitrile (Merck, 1,499,230–935)/water solution (2:2:1, v/v), vortex-mixed, and subjected to low-temperature ultrasonication for 30 min. The mixture was incubated at −20 °C for 10 min, followed by centrifugation at 14,000 × g for 20 min (4 °C) in a precooled high-speed centrifuge (Eppendorf 5430R). The supernatant was vacuum-dried and reconstituted in 100 μL of acetonitrile/water solution (1:1, v/v) for mass spectrometry analysis. After vortexing, the solution was centrifuged at 14,000 × g (4 °C) for 15 min, and the supernatant was collected for instrumental analysis.

Quality control (QC) samples were prepared by pooling equal volumes of all test samples. These QC samples were used to assess instrument stability prior to injection, equilibrate the chromatography-mass spectrometry system, and evaluate overall system stability throughout the experimental process.

#### 2.5.2 LC–MS analysis

In the chromatographic analysis experiment, an Agilent 1,290 Infinity LC ultra–high-performance liquid chromatography system equipped with a hydrophilic interaction chromatography column (HILIC) was used to complete the component separation. The analysis conditions were set as follows: the column temperature was maintained at a constant 25 °C, the flow rate of the mobile phase was controlled at 0.5 mL/min, and the single injection volume was 2 μL. The mobile phase consisted of A (an aqueous solution containing 25 mM ammonium acetate and 25 mM ammonia) and B (acetonitrile), and a gradient elution program was adopted: in the initial stage (0–0.5 min), the proportion of acetonitrile was maintained at 95%; from 0.5–7 min, the proportion of acetonitrile linearly decreased to 65%; from 7 to 8 min, it further decreased to 40% and was maintained until 9 min; from 9 to 9.1 min, it rapidly increased back to 95%, and finally, from 9.1 to 12 min, a high proportion of acetonitrile was maintained to equilibrate the system. Throughout the process, the samples were placed in an autosampler at 4 °C to eliminate the influence of temperature fluctuations. A random injection sequence was used to eliminate the interference of instrument signal drift, and quality control samples (QC) were inserted at intervals to monitor the system stability and data reliability.

For mass spectrometry detection, an AB Triple TOF 6600 high–resolution mass spectrometer was used to simultaneously acquire full–scan first-order spectra (TOF MS) and data-dependent second-order fragment ion spectra (IDA) in both positive and negative ion modes. The working parameters of the electrospray ionization source (ESI) were optimized as follows: the nebulizing gas (Gas1/Gas2) was set at 60 psi, the curtain gas (CUR) was 30 psi, the ion source temperature was 600 °C, and the floating voltage (ISVF) was ±5,500 V. The mass spectrometry scanning range was set as: for the first-order mass spectrometry, m/z was from 60 to 1,000 Da (accumulation time 0.20 s/spectrum), and for the second-order mass spectrometry, m/z was from 25 to 1,000 Da (accumulation time 0.05 s/spectrum). The second-order mass spectrometry adopted the high-sensitivity mode, with the collision energy set at 35 ± 15 eV, the declustering potential (DP) at ±60 V, and a dynamic exclusion algorithm was configured (to exclude isotopic peaks within a 4-Da window), and 10 candidate ions were monitored per acquisition cycle.

#### 2.5.3 Data processing

After the initial dataset was converted to the standard. MzML format using the ProteoWizard tool, the XCMS software was employed for chromatographic peak alignment, retention time standardization correction, and ion peak area integration. The parameter configuration of the XCMS algorithm was as follows: during the peak detection phase, the mass deviation tolerance was set to 10 ppm, the scanning peak width range was limited to 10–60 acquisition points, and the signal intensity pre-filtering threshold was set to 10–100 times the baseline noise; in the peak grouping process, a 5-s retention time window was used, the mass tolerance width was 0.025 Da, and it was required that the characteristic peak should be present in at least 50% of the samples.

When conducting quality control on the extracted metabolomic data, a completeness screening was first carried out, and metabolite features with a missing ratio of more than 50% within the group were removed. The K-Nearest Neighbors (KNN) algorithm was used to fill in the remaining missing values. Extreme outliers were removed based on the three-standard-deviation rule. Finally, normalization was performed on the total ion current chromatographic peak area to ensure the comparability of the quantitative data between samples and metabolites.

## 3 Results

### 3.1 Comparative analysis of nutrient composition in cold season and warm season forages

As shown in Table 1, warm-season forages exhibited significantly greater (*p* < 0.01) concentrations of dry matter (DM), crude protein (CP), and ether extract (EE). Conversely, cold-season forages were characterized by significantly higher (*p* < 0.01) levels of neutral detergent fiber (NDF) and acid detergent fiber (ADF).

**Table 1:** Comparison of nutritional components of forage during warm and cold seasons.

Item	Groups		*p*-value
	Warm season	Cold season	
DM (%)	94.50^a^ ± 0.36	86.78^b^ ± 0.28	<0.001
CP (%)	21.25^a^ ± 0.47	6.27^b^ ± 0.14	<0.001
EE (%)	2.56^a^ ± 0.13	0.54^b^ ± 0.08	<0.001
NDF (%)	53.15^a^ ± 0.36	57.26^b^ ± 0.75	0.004
ADF (%)	31.48^a^ ± 0.47	35.37^b^ ± 1.46	0.03

Different superscript letters within the same row indicate statistically significant differences (*p* < 0.05), while shared letters denote no significant variation (*p* > 0.05).

### 3.2 Comparative analysis of serum biochemical indices of Tibetan sheep in cold and warm seasons

As shown in Table 2, the concentrations of alanine transaminase (ALT), total cholesterol (TC), creatinine (CRE), and blood urea nitrogen (BUN) in the serum of Tibetan sheep during the warm season were significantly higher than those during the cold season (*p* < 0.05). No significant differences were observed in the concentrations of glutamate oxaloacetate transaminase (GOT), total protein (TP), albumin (ALB), globulin (GLB), and triglyceride (TG) between the cold and warm seasons.

**Table 2:** Comparison of serum biochemical parameters in Tibetan sheep during warm and cold seasons.

Item	Groups		*p*-value
	Warm season	Cold season	
GOT (U/L)	31.71 ± 2.39	29.97 ± 1.95	0.300
ALT (U/L)	12.66^a^ ± 0.58	6.95^b^ ± 0.66	<0.001
TP (g/L)	7.95 ± 0.37	7.41 ± 0.59	0.320
ALB (g/L)	4.85 ± 0.22	4.61 ± 0.40	0.340
GLB (g/L)	3.15 ± 0.68	2.87 ± 0.27	0.600
TC (mmol/L)	1.28^a^ ± 0.13	0.90^b^ ± 0.02	0.030
TG (g/L)	0.14 ± 0.01	0.15 ± 0.01	0.490
CRE (μmol/mL)	61.85^a^ ± 2.96	51.01^b^ ± 3.23	0.030
BUN (g/L)	0.44^a^ ± 0.03	0.15^b^ ± 0.01	<0.001

### 3.3 Comparative analysis of the composition and function of Tibetan sheep gut microbiota between cold and warm seasons

#### 3.3.1 Sequencing data processing and evaluation

Shotgun metagenomic sequencing was performed on six rectal content samples (*n* = 3 per season). After quality control, all six libraries showed valid read ratios above 98%. Clean reads were subsequently assembled *de novo*, and the resulting contigs showed N50 values ranging from 940 to 1,070 bp. Detailed per-sample sequencing quality-control statistics are provided in Table S1.

#### 3.3.2 Alpha diversity analysis

**Figure 1:**
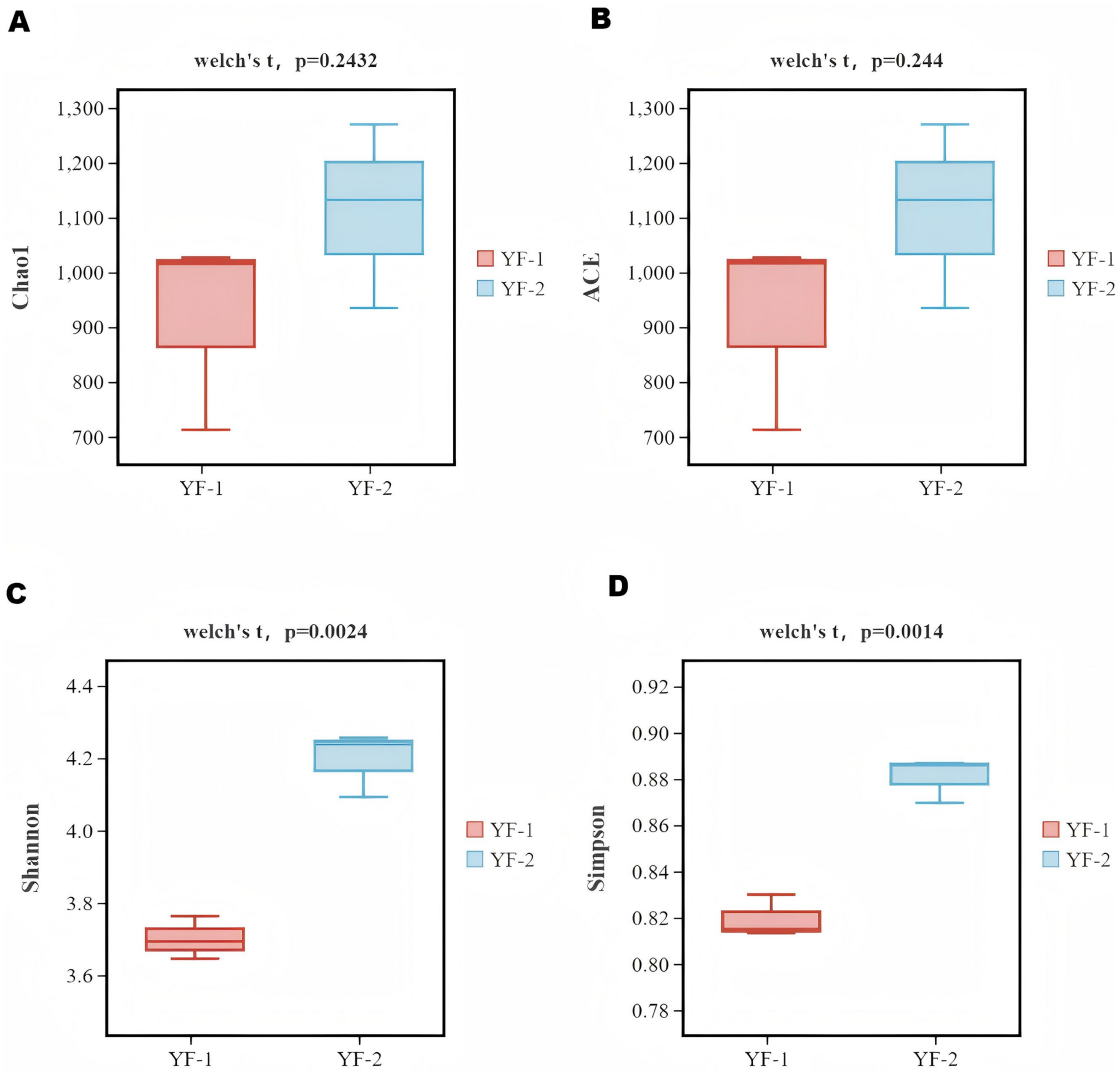
Results of species ɑ diversity analysis **(A–D)**. Chao1 index **(A)**, ACE index **(B)**, Shannon index **(C)**, and Simpson index **(D)**.

Results of species *α*-diversity index analysis (at the genus level) showed (Figure 1) that the Shannon and Simpson indices in the YF-1 group were significantly lower than those in the YF-2 group (*p* < 0.01). The cold and warm seasons had no significant effects on the Chao 1 and ACE indices of rectal microbiota in the two groups of Tibetan sheep (*p* > 0.05), indicating that the community diversity and dominant species diversity of gut microbiota in Tibetan sheep during the cold season were significantly higher than those during the warm season.

#### 3.3.3 Beta diversity analysis

**Figure 2:**
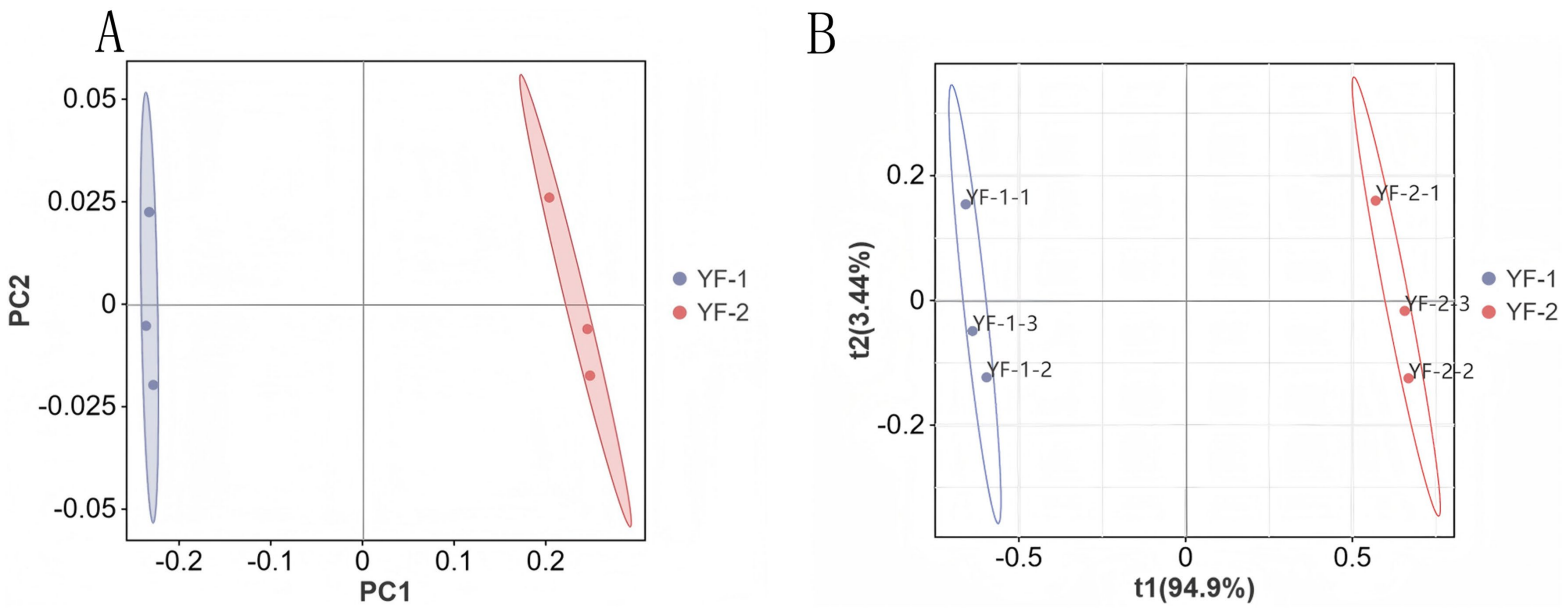
Species β-diversity analysis results: principal component analysis (PCA) **(A)** and partial least squares discriminant analysis (PLS-DA) **(B)**.

Principal component analysis (PCA) results indicated (Figure 2A) that samples from the YF-1 and YF-2 groups exhibited substantial dispersion, suggesting differences in the gut microbial composition of Tibetan sheep between cold and warm seasons. Partial least squares discriminant analysis (PLS-DA) results (Figure 2B) revealed minimal intra-group variation but pronounced inter-group separation, with good data dispersion. This confirmed significant differences in rectal microbiota between cold and warm seasons and validated the suitability of the dataset for subsequent analysis.

#### 3.3.4 Species annotation analysis

**Figure 3:**
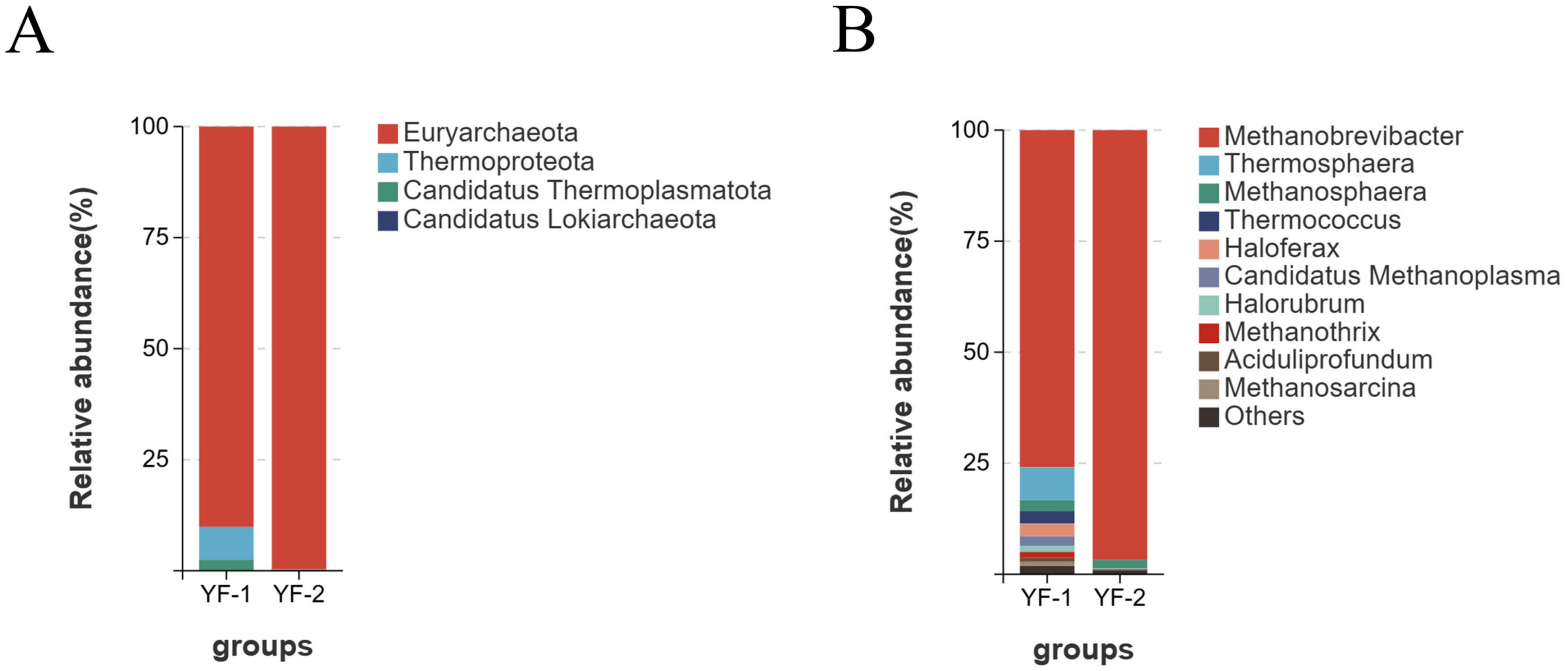
Translation of the relative abundance of the phylum **(A)** and genus **(B)** levels of archaea in the intestines of Tibetan sheep during cold and warm seasons.

Across the six metagenomic samples, archaeal sequences were assigned to 4 phyla, 7 classes, 11 orders, 13 families, 24 genera, and 94 species. Significant differences in gut archaeal composition were observed between cold and warm seasons at the phylum and genus levels. As shown in Figure 3, the dominant archaeal phyla in Tibetan sheep during the warm (YF-1) and cold (YF-2) seasons included Euryarchaeota (YF-1: 90.14%, YF-2: 99.69%), Thermoproteota (YF-1: 7.37%, YF-2: 0.01%), Candidatus Thermoplasmatota (YF-1: 2.48%, YF-2: 0.20%), and Candidatus Lokiarchaeota (YF-1: 0%, YF-2: 0.10%). Euryarchaeota was the most abundant phylum in both YF-1 and YF-2, followed by Thermoproteota, indicating shared compositional commonalities in gut archaeal communities across seasons despite relative abundance shifts.

**Figure 4:**
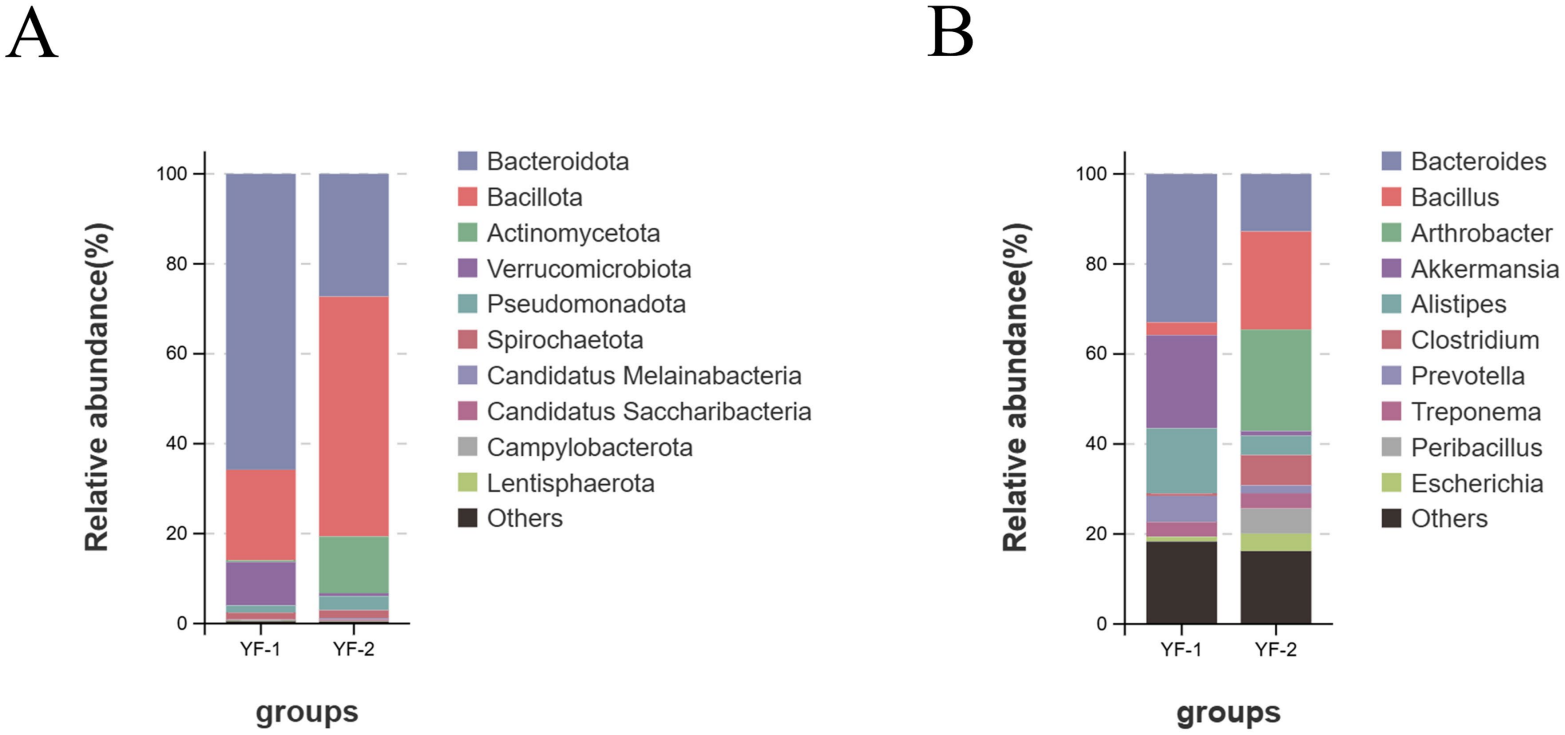
The relative abundance of bacterial phylum **(A)** and genus **(B)** in the intestines of Tibetan sheep during cold and warm seasons.

At the bacterial level, a total of 103 phyla, 90 classes, 174 orders, 405 families, 1,384 genera, and 5,051 species were annotated with significant compositional differences observed between cold and warm seasons. As shown in Figure 4, the top 10 bacterial phyla by relative abundance in Tibetan sheep gut microbiota during warm (YF-1) and cold (YF-2) seasons included Bacteroidota (YF-1: 58.22%, YF-2: 24.31%), Bacillota (formerly Firmicutes, YF-1: 17.82%, YF-2: 47.47%), Actinomycetota (YF-1: 0.33%, YF-2: 11.22%), Verrucomicrobiota (YF-1: 8.55%, YF-2: 0.60%), Pseudomonadota (formerly Proteobacteria, YF-1: 1.41%, YF-2: 2.79%), Spirochaetota (YF-1: 1.36%, YF-2: 1.52%), Candidatus Melainabacteria (YF-1: 0.08%, YF-2: 0.28%), Candidatus Saccharibacteria (YF-1: 0.01%, YF-2: 0.33%), Campylobacterota (YF-1: 0.16%, YF-2: 0.02%), and Lentisphaerota (YF-1: 0.06%, YF-2: 0.07%). Dominant phyla across both seasons included Bacteroidota, Bacillota, Actinomycetota, and Verrucomicrobiota. Specifically, Bacteroidota and Verrucomicrobiota showed significantly higher relative abundances in warm-season samples (YF-1) compared to cold-season samples (YF-2) (*p* < 0.05), whereas Bacillota and Actinomycetota exhibited the opposite trend (*p* < 0.05). In the gut bacteria of Tibetan sheep during the warm season, the dominant genera compared to the cold season were *Bacteroides* (YF-1: 12.55%, YF-2: 5.47%), *Akkermansia* (YF-1: 7.87%, YF-2: 0.45%), and *Alistipes* (YF-1: 5.51%, YF-2: 1.83%). In contrast, the relative abundances of *Bacillus* (YF-1: 1.06%, YF-2: 9.32%) and *Arthrobacter* (YF-1: 0.01%, YF-2: 9.65%) in the cold season were significantly higher than those in the warm season (*p* < 0.05).

**Figure 5:**
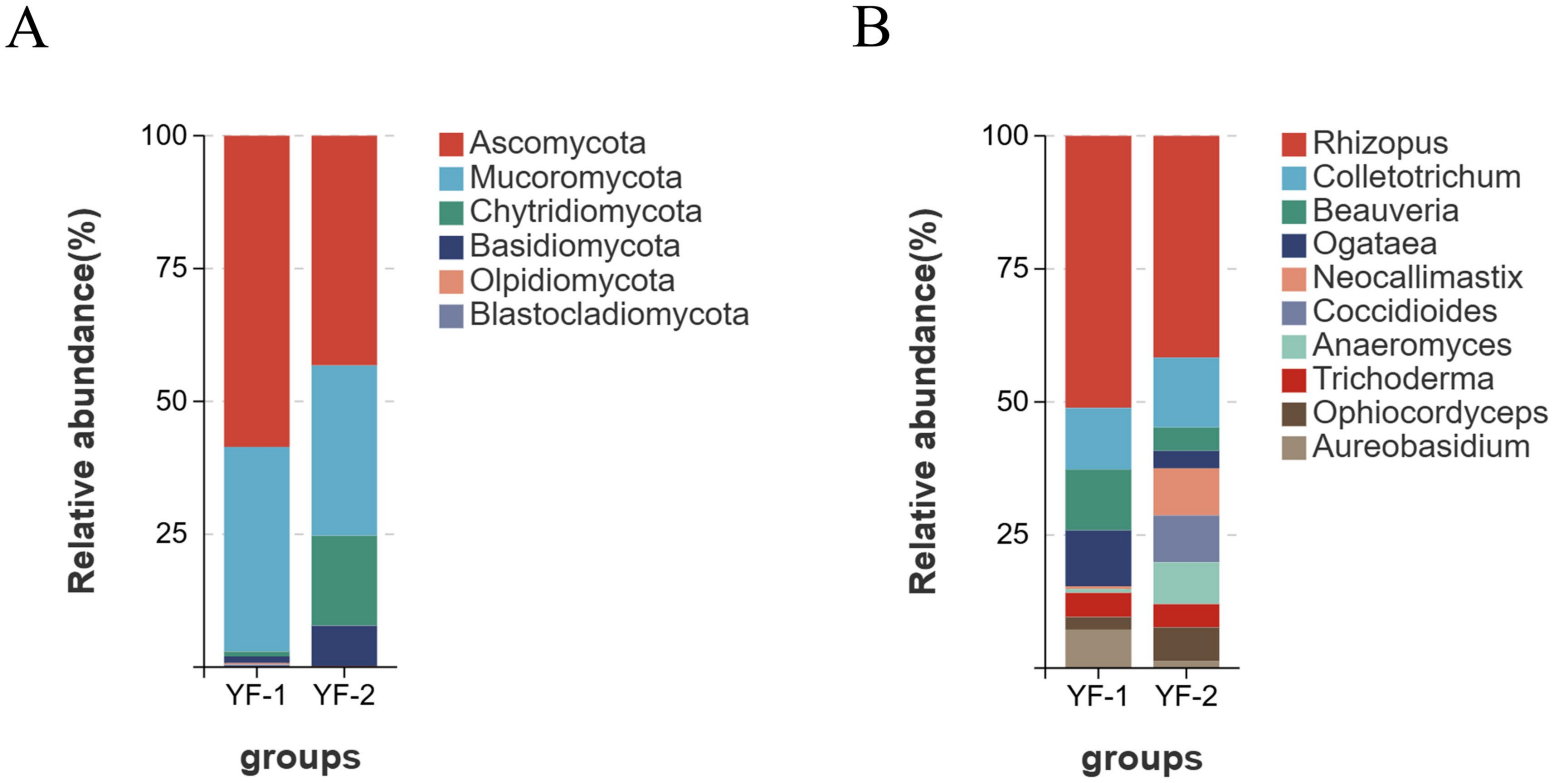
The relative abundance of intestinal fungi in Tibetan sheep at the phylum **(A)** and genus **(B)** levels during cold and warm seasons.

At the fungal level, a total of 6 phyla, 17 classes, 30 orders, 48 families, 56 genera, and 74 species were annotated with significant compositional differences observed between cold and warm seasons. As shown in Figure 5, shared dominant fungal phyla across seasons included Ascomycota (YF-1: 58.56%, YF-2: 43.24%) and Mucoromycota (YF-1: 38.51%, YF-2: 32.03%). At the genus level, 31 genera were shared between seasons, with 9 genera unique to the warm season (YF-1) and 16 unique to the cold season (YF-2). The top 5 dominant genera showed consistent trends across seasons: *Rhizopus* (YF-1: 30.41%, YF-2: 31.46%), *Colletotrichum* (YF-1: 7.78%, YF-2: 9.91%), *Beauveria* (YF-1: 7.70%, YF-2: 3.32%), *Ogataea* (YF-1: 7.08%, YF-2: 2.47%), and *Neocallimastix* (YF-1: 0.34%, YF-2: 6.69%). Cold-season samples additionally featured *Coccidioides* (YF-1: 0%, YF-2: 6.67%) and *Anaeromyces* (YF-1: 0.49%, YF-2: 5.91%) as dominant genera.

#### 3.3.5 Analysis of differential intestinal flora in Tibetan sheep during cold and warm seasons

**Figure 6:**
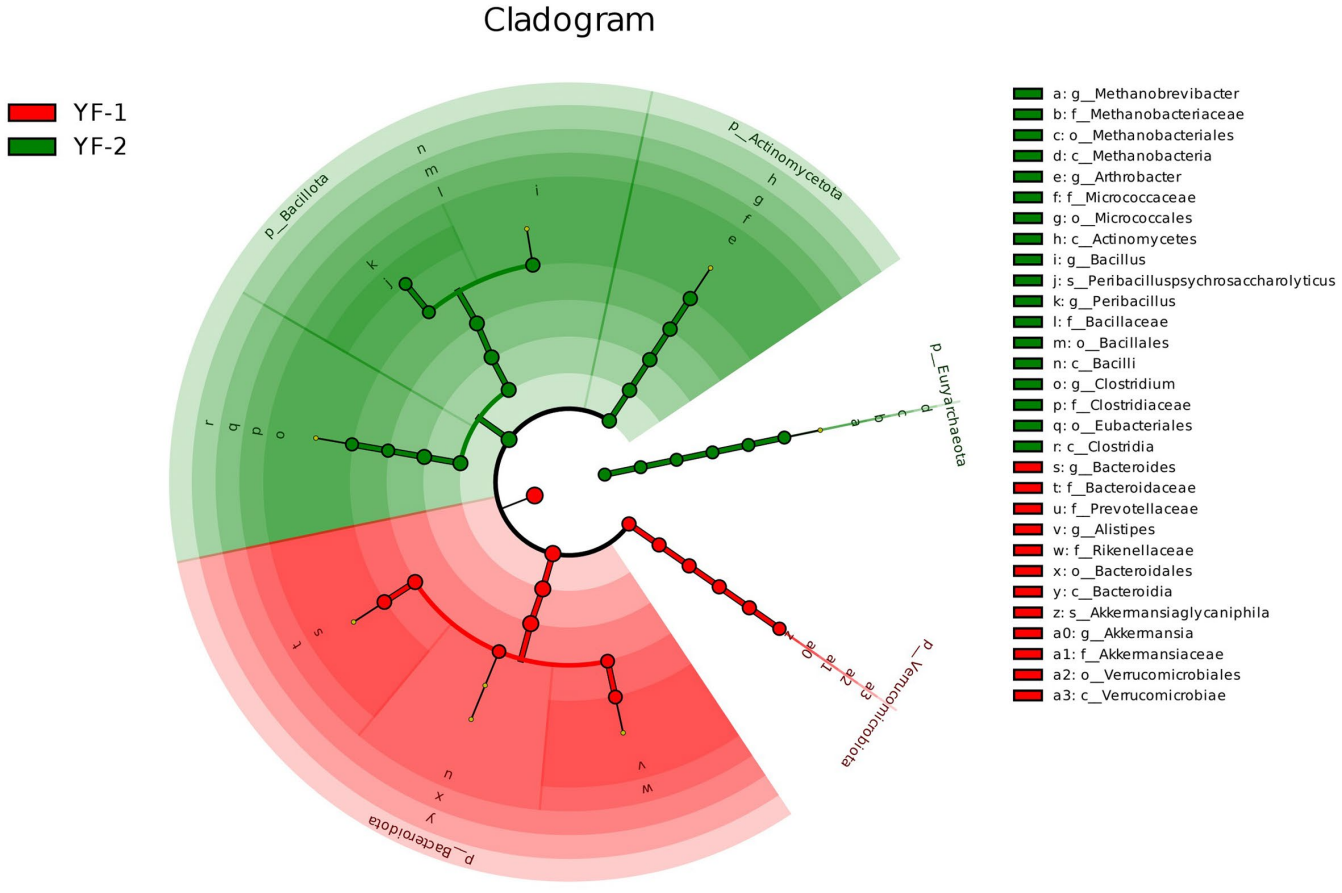
The branch evolution diagram of LEfSe analysis for the intestinal microbiota of Tibetan sheep during cold and warm seasons.

**Figure 7:**
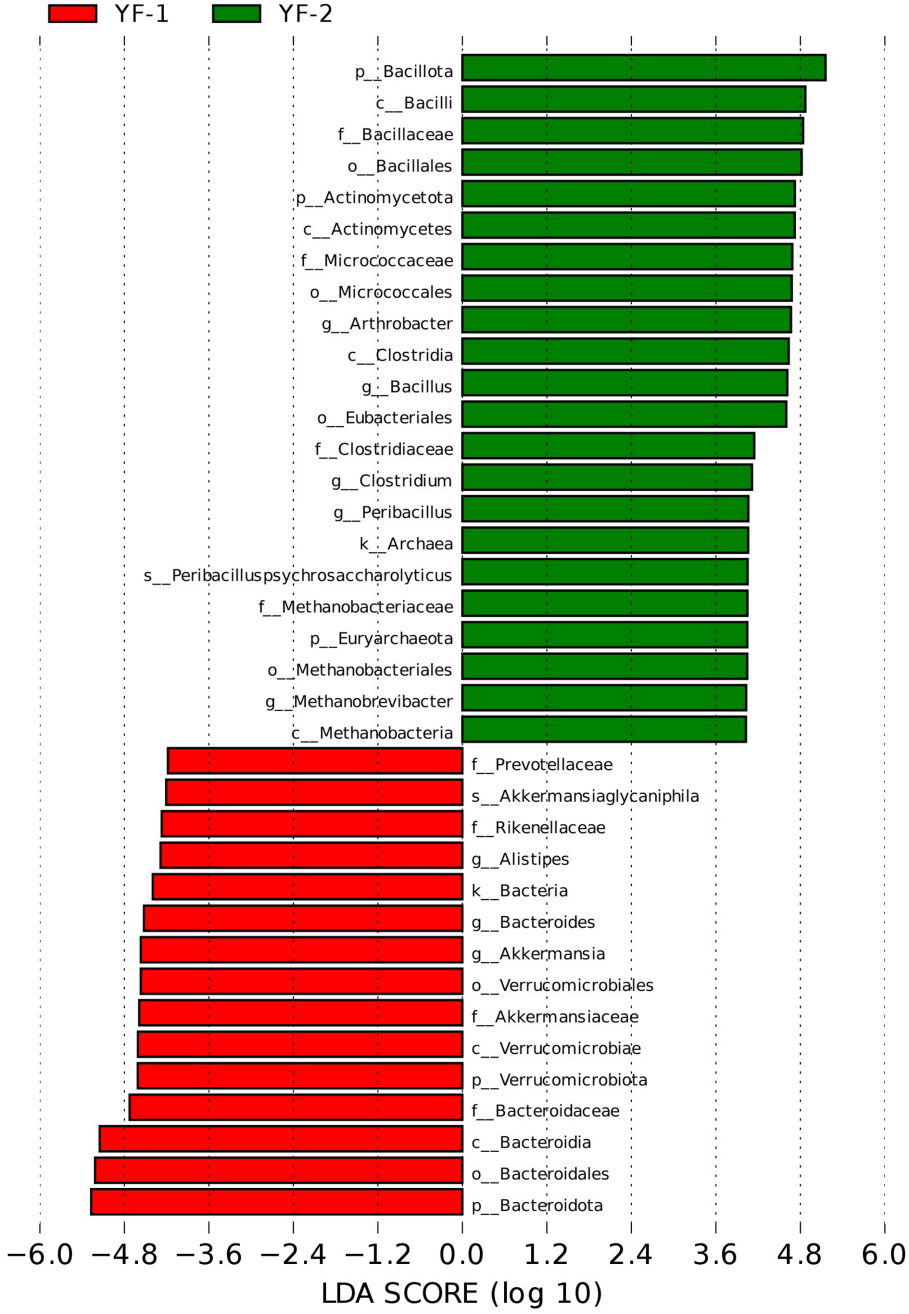
The bar chart of LDA score distribution from LEfSe analysis of the intestinal microbiota in Tibetan sheep during cold and warm seasons.

LEfSe analysis (Figures 6, 7) further identified differential gut microbiota between cold and warm seasons in Tibetan sheep, with statistically significant biomarkers (LDA score > 4.3, *p* < 0.05) visualized and analyzed. A total of 37 discriminative microbial taxa were identified, including 15 enriched in the warm season: Bacteroidota (phylum), Bacteroidia (class), Rikenellaceae (family), Akkermansiaceae (family), Verrucomicrobiota (phylum), *Bacteroides* (genus), *Alistipes* (genus), Verrucomicrobiales (order), Bacteroidales (order), *Akkermansia* (genus), Prevotellaceae (family), Bacteroidaceae (family), *Akkermansiaglycaniphila* (species), and Verrucomicrobiae (class). Cold-season samples harbored 22 discriminative taxa: *Arthrobacter* (genus), Actinomycetes (class), *Peribacillus* (genus), *Clostridium* (genus), Clostridiaceae (family), Methanobacteriaceae (family), Methanobacteria (class), Micrococcaceae (family), Methanobacteriales (order), Clostridia (class), Bacillales (order), Eubacteriales (order), Actinomycetota (phylum), Bacilli (class), Micrococcales (order), Bacillota (phylum), Bacillaceae (family), *Peribacilluspsychrosaccharolyticus* (species), *Methanobrevibacter* (genus), *Bacillus* (genus), and Euryarchaeota (phylum).

#### 3.3.6 Functional analysis of intestinal flora in Tibetan sheep during cold and warm seasons

##### 3.3.6.1 Egg NOG functional annotation analysis

**Figure 8:**
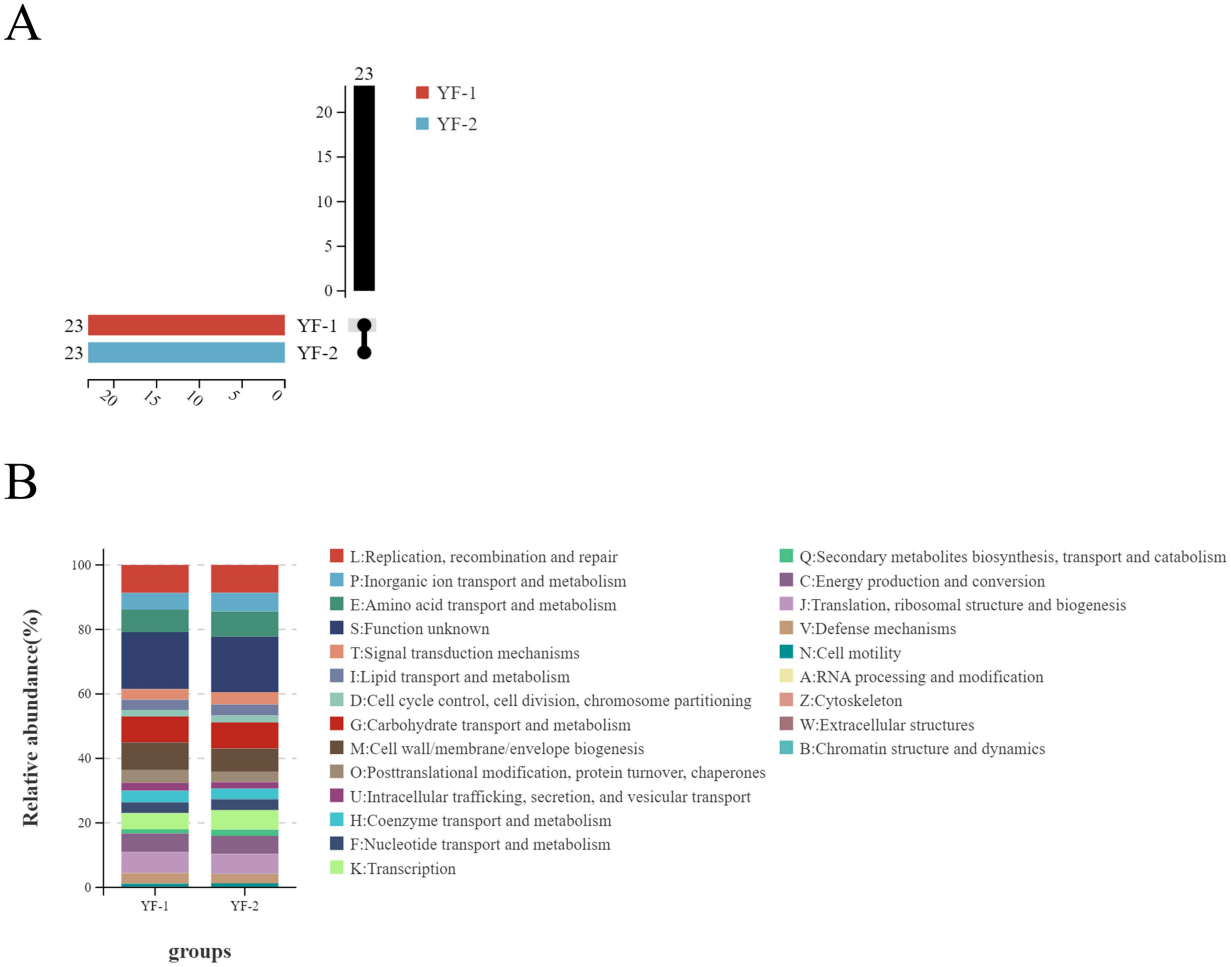
Functional annotation analysis of the intestinal microbiota in Tibetan sheep during cold and warm seasons using eggNOG: **(A)** Upset diagram, **(B)** functional distribution stacked bar chart.

Functional annotation of gut microbiota genes from cold and warm seasons was performed using the egg NOG database (Figure 8). Results showed that genes were classified into 23 egg NOG functional pathways with similar abundances and minimal differences between seasons. The highest number of genes were annotated to Replication, recombination and repair, followed by Carbohydrate transport and metabolism, Cell wall/membrane/envelope biogenesis, Amino acid transport and metabolism, Translation, ribosomal structure and biogenesis, Energy production and conversion, and Transcription.

##### 3.3.6.2 KEGG functional enrichment analysis

**Figure 9:**
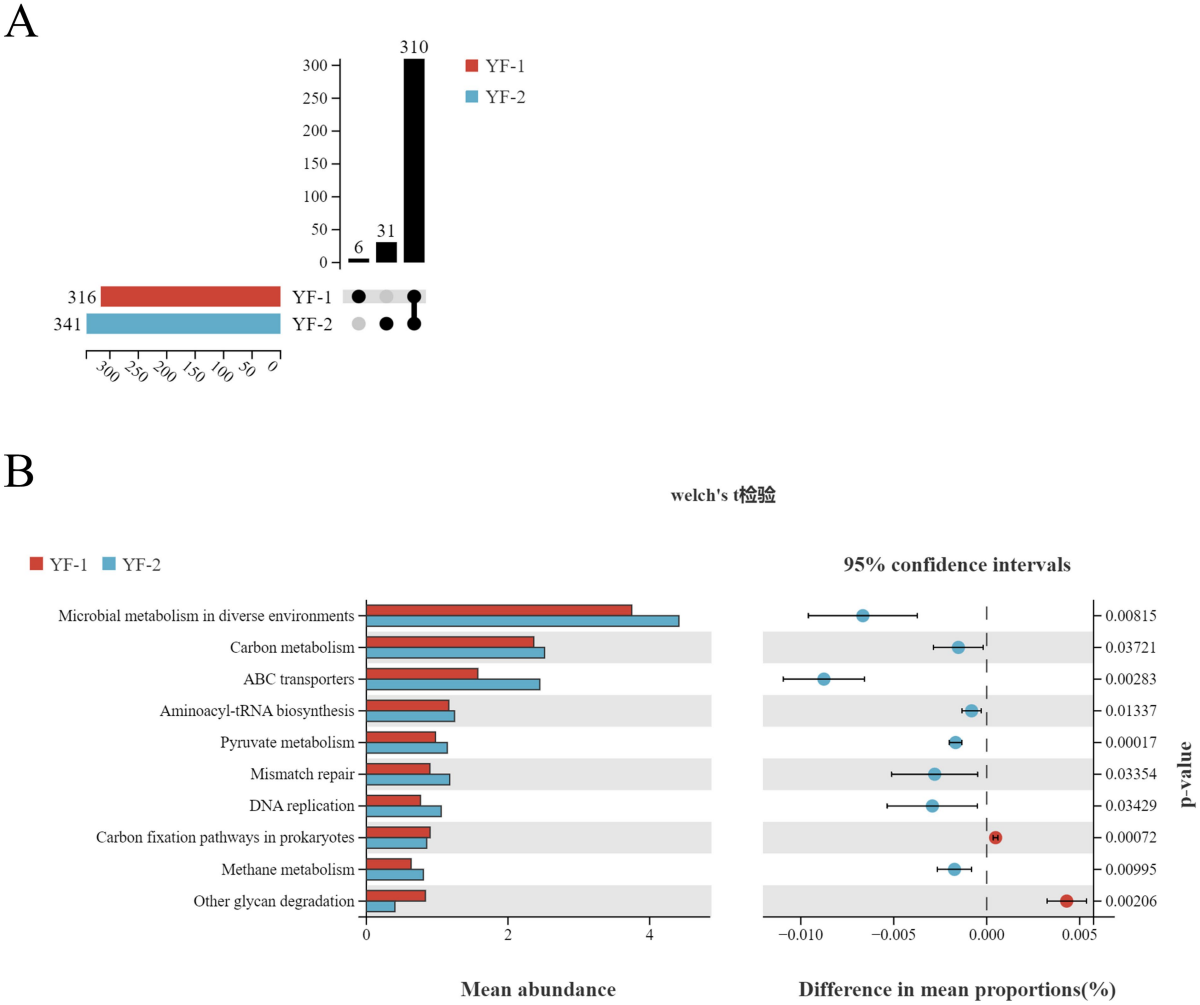
KEGG functional enrichment analysis of intestinal microbiota in Tibetan sheep during cold and warm seasons: unique and shared KEGG level 3 pathways between YF-1 and YF-2 **(A)**, and significantly differential KEGG level 3 pathways between the two groups **(B)**.

Differentially expressed genes in gut microbiota of Tibetan sheep during warm and cold seasons were significantly enriched in 316 and 341 KEGG level 3 pathways, respectively, with 6 pathways exclusive to the warm season and 31 exclusive to the cold season (Figure 9A). Further analysis identified 10 statistically significant metabolic pathways at the KEGG level 3 between seasons (Figure 9B). Specifically, warm-season microbiota showed marked enrichment in Carbon fixation pathways in prokaryotes and Other glycan degradation. Conversely, cold-season microbiota exhibited significant increases in Microbial metabolism in diverse environments, Carbon metabolism, ABC transporters, Aminoacyl-tRNA biosynthesis, Pyruvate metabolism, Mismatch repair, DNA replication, and Methane metabolism.

### 3.4 Comparative analysis of the composition and function of metabolites in the gut microbiota of Tibetan sheep in cold and warm seasons

#### 3.4.1 Multivariate statistical analysis of metabolites in the gut microbiota of Tibetan sheep in cold and warm seasons

**Figure 10:**
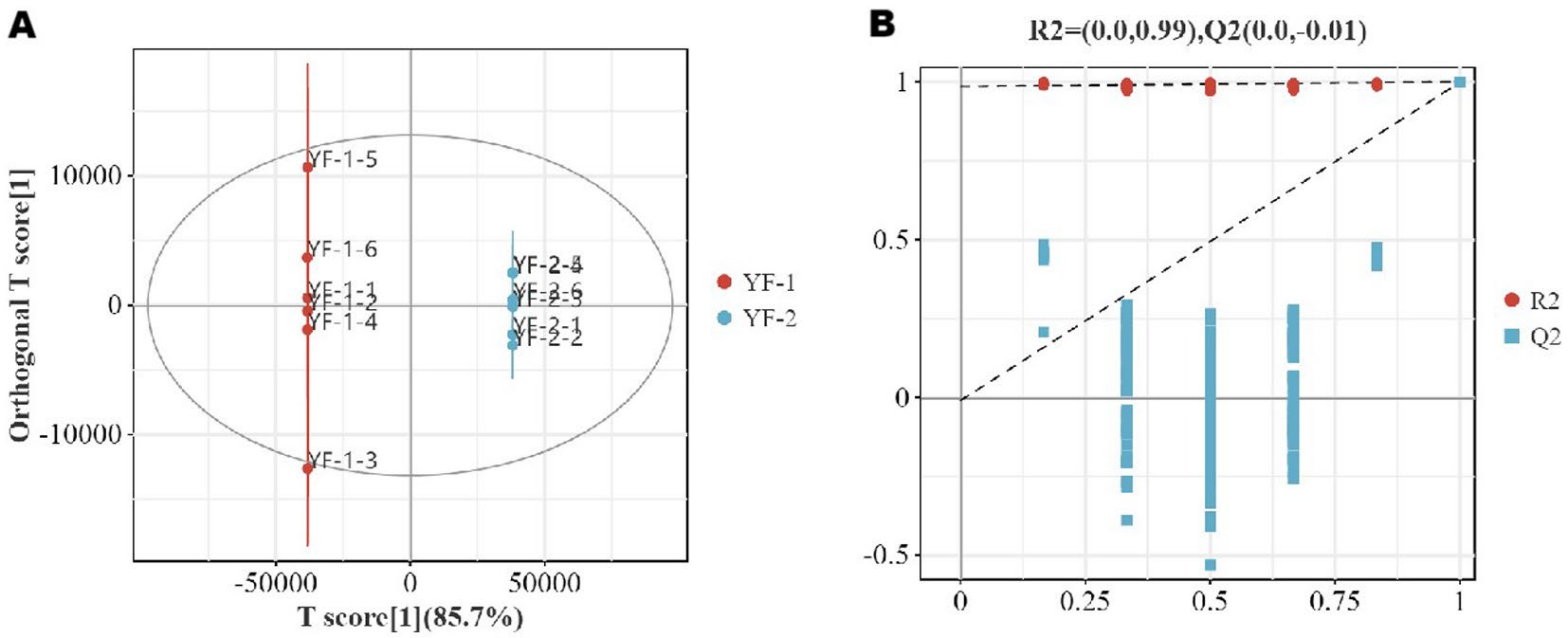
Plot of OPLS-DA scores **(A)** and substitution test **(B)** in the positive ion mode.

**Figure 11:**
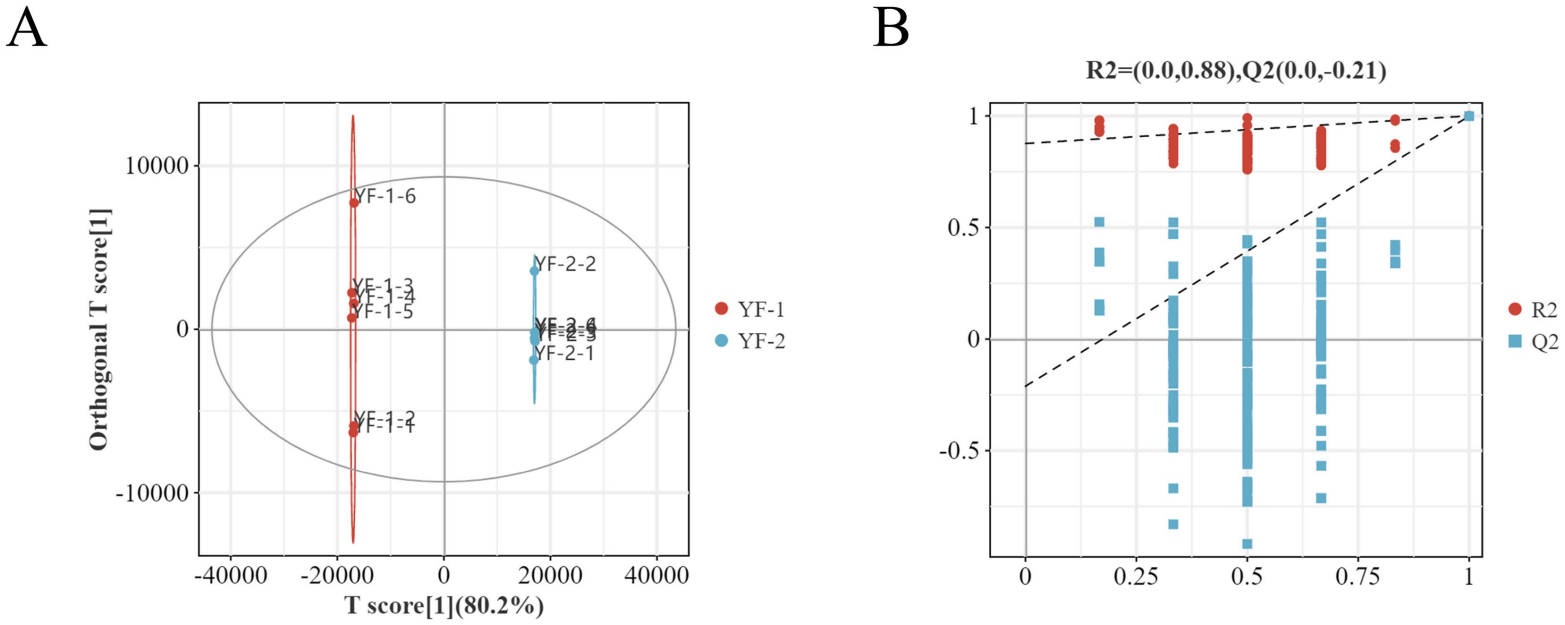
Plot of OPLS-DA scores **(A)** and substitution test **(B)** in the negative ion mode.

Part of the untargeted metabolomics dataset analyzed in this section was derived from the same sample cohort used in our previous metabolomics-focused study; however, all analyses, result organization, and figure generation presented here were re-performed under the distinct objective of cross-omics integration in the current manuscript. Orthogonal partial least squares discriminant analysis (OPLS-DA) models (Figures 10A, 11A) and permutation test plots (Figures 10B, 11B) were applied to characterize metabolite differences in the gut microbiota of Tibetan sheep between cold and warm seasons. As shown, warm-season and cold-season metabolite samples clustered into distinct confidence intervals with minimal intra-group variation and pronounced inter-group separation. All *R*^2^ values approached 1, with *R*^2^ > *Q*^2^ and *Q*^2^ regression line intercepts <0, indicating no overfitting and valid model performance for accurate sample representation. OPLS-DA confirmed significant metabolic differences between seasons, and permutation tests further validated the model’s robustness.

#### 3.4.2 Differential analysis of metabolites in the gut microbiota of Tibetan sheep between cold and warm seasons

**Figure 12:**
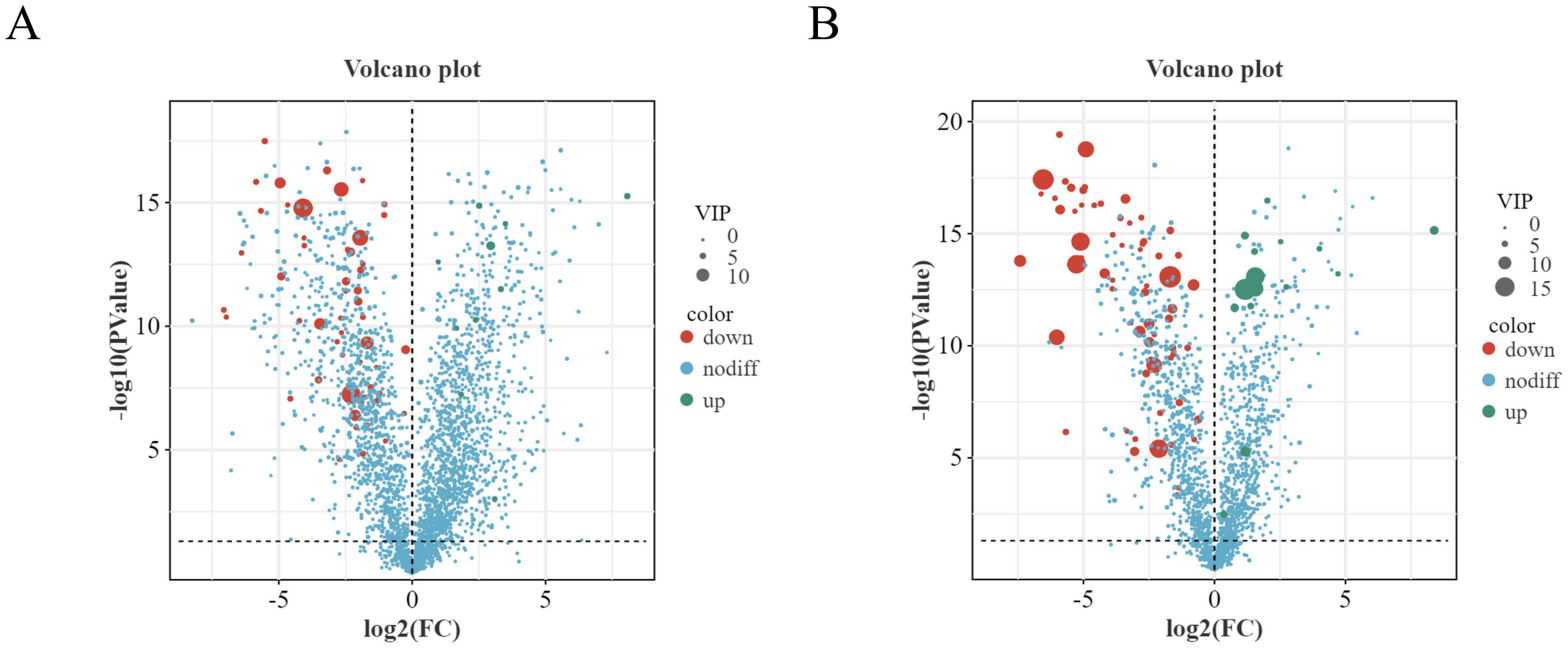
Volcano plot of differential metabolites in the intestines of Tibetan sheep during warm and cold seasons under positive **(A)** and negative **(B)** ion modes.

**Figure 13:**
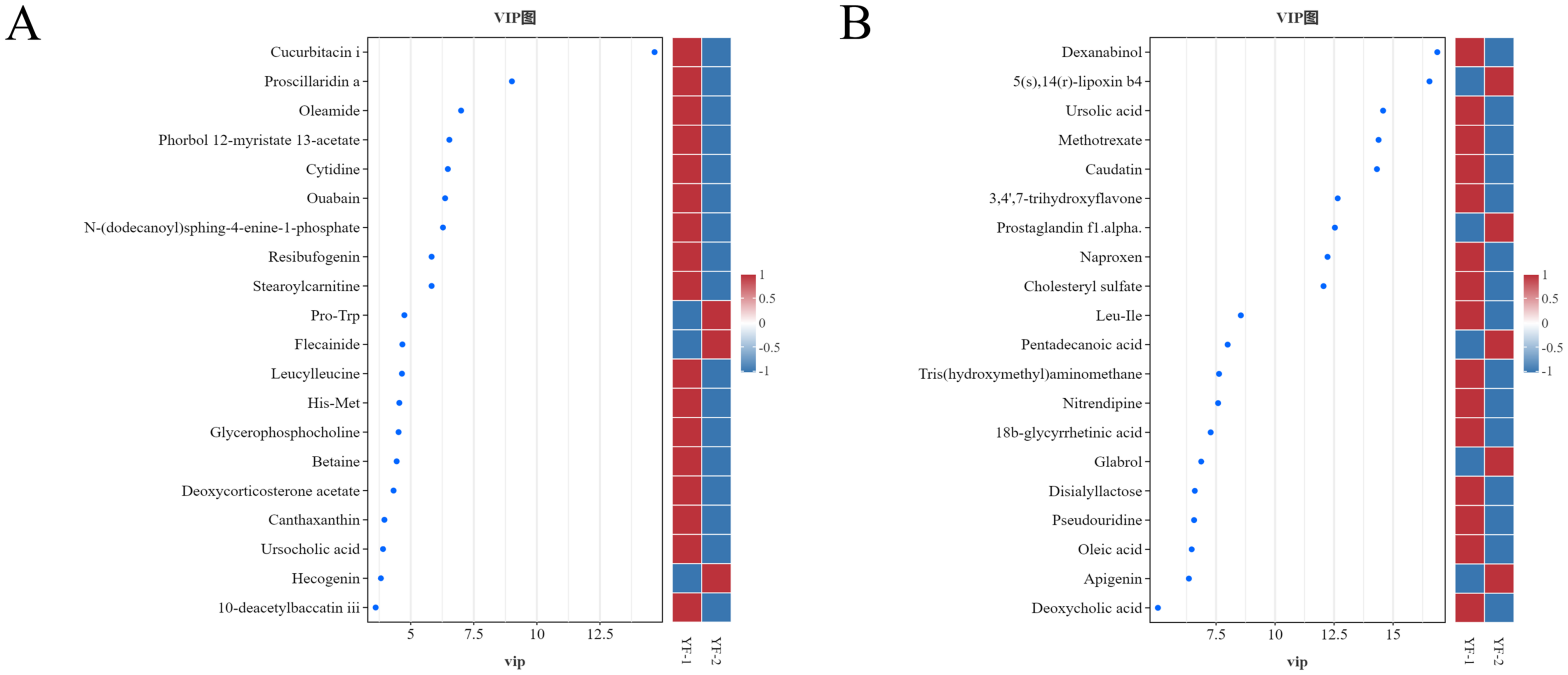
VIP plot of differential metabolites in the intestines of Tibetan sheep during warm and cold seasons under positive **(A)** and negative **(B)** ion modes.

Based on OPLS-DA results combined with VIP > 3, univariate *T*-test *p* < 0.05, and |log₂|FC ≥ 1 or ≤ 1, differential metabolites detected under positive and negative ion modes were analyzed using volcano plots (Figure 12) and VIP plots (Figure 13). A total of 476 and 383 differential metabolites were identified in cold and warm seasons under positive and negative ion modes respectively: 148 upregulated and 328 downregulated in positive ion mode, 135 upregulated and 248 downregulated in negative ion mode. Analysis of top 10 significantly differential microbiota metabolites based on logarithmic fold changes revealed: under positive ion mode, Flecainide, Desipramine, Milnacipran, Ginkgolide A, 4-androstene-3,17-dione, N6-methyl-2′-deoxyadenosine, Citrinin, Melatonin, 4-hydroxybenzaldehyde, and His-Asp were significantly upregulated in cold-season samples (*p* < 0.05), while Teniposide, Gomisin C, Pro-Pro, 4-aminobiphenyl, Sulthiame, Clofazimine, His-Met, Chloramphenicol, Leucylleucine, and Val-Val showed marked upregulation in warm-season samples (*p* < 0.05). Under negative ion mode, Glabrol, N-acetyl-L-phenylalanine, 2′-deoxyguanosine 5′-monophosphate, 2,2-dimethylglutaric acid, 6-hydroxyhexanoate, Caproic acid, Butanoic acid, Prostaglandin E2, Hexadecanedioic acid, and 3-(3-Hydroxyphenyl)propanoic acid were significantly upregulated in cold-season samples (*p* < 0.05), whereas Harmaline, Naproxen, Pro-Thr, Pseudouridine, Luteolin 7-glucoside, Leu-Leu, Ursolic acid, Caudatin, Astragalin, and Prostaglandin I2 demonstrated marked upregulation in warm-season samples (*p* < 0.05).

Additionally, further screening analysis of differential metabolites was performed by ranking based on VIP values, where higher VIP values indicated greater importance. Specifically under positive ion mode, Pro-Trp and Glycoursodeoxycholic acid were significantly upregulated in cold-season samples (*p* < 0.05), while Cucurbitacin I, Stearoylcarnitine, and Leucyl-leucine showed marked upregulation in warm-season samples (*p* < 0.05). Under negative ion mode, 5(S),14(R)-lipoxin B4, Prostaglandin F1α, and Pentadecanoic acid were significantly upregulated in cold-season samples (*p* < 0.05), whereas Ursolic acid, Methotrexate, Cholesteryl sulfate, 18β-glycyrrhetinic acid, Oleic acid, and Deoxycholic acid demonstrated marked upregulation in warm-season samples (*p* < 0.05).

#### 3.4.3 Differential pathway analysis of metabolites in the gut microbiota of Tibetan sheep between cold and warm seasons

**Figure 14:**
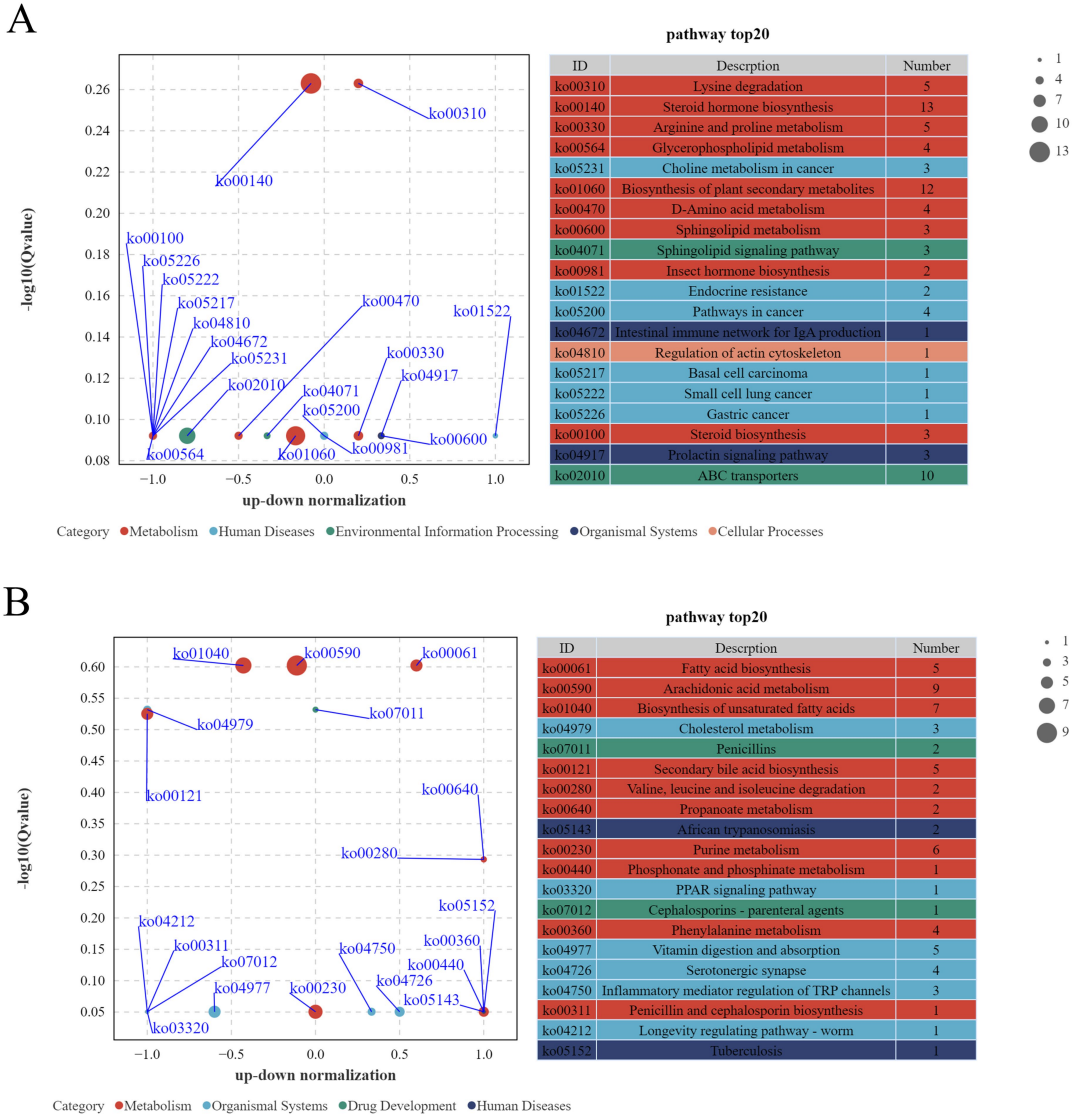
Bubble plot of differential enrichment of intestinal metabolites in Tibetan sheep under positive **(A)** and negative **(B)** ion modes.

**Figure 15:**
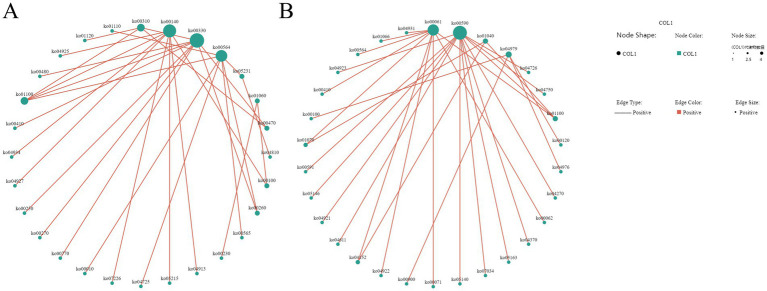
Differential enrichment network diagram of intestinal metabolites in Tibetan sheep under positive **(A)** and negative **(B)** ion modes.

KEGG Pathway functional annotation of differential metabolites revealed 121 and 110 metabolites annotated to KEGG pathways under positive and negative ion modes, respectively. Visualization was performed using differential enrichment bubble plots (Figure 14) and network diagrams (Figure 15), where larger -log₁₀(pvalue) indicated stronger significance, and dot size represented the number of differential metabolites enriched in respective pathways. Positive ion mode metabolites showed significant enrichment in Lysine degradation, Steroid hormone biosynthesis, Arginine and proline metabolism, and Glycerophospholipid metabolism. Negative ion mode metabolites were significantly enriched in Fatty acid biosynthesis, Arachidonic acid metabolism, Biosynthesis of unsaturated fatty acids, Cholesterol metabolism, Secondary bile acid biosynthesis, and Propanoate metabolism.

### 3.5 Integrative multi-omics analysis of the gut microbiota and metabolites in Tibetan sheep in cold and warm seasons

#### 3.5.1 O2PLS model analysis

**Figure 16:**
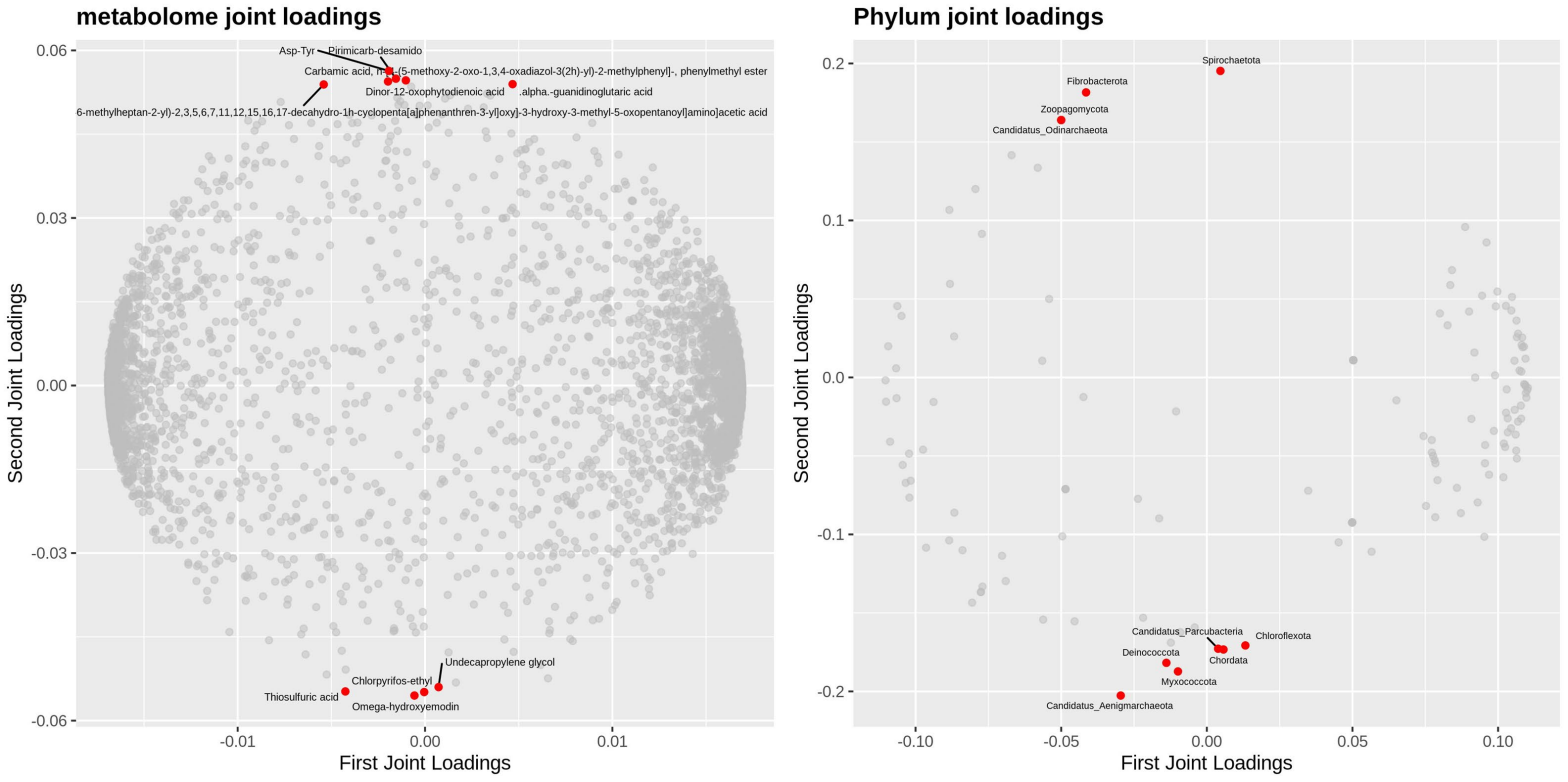
O_2_PLS loading plot of two omics datasets at the phylum level.

**Figure 17:**
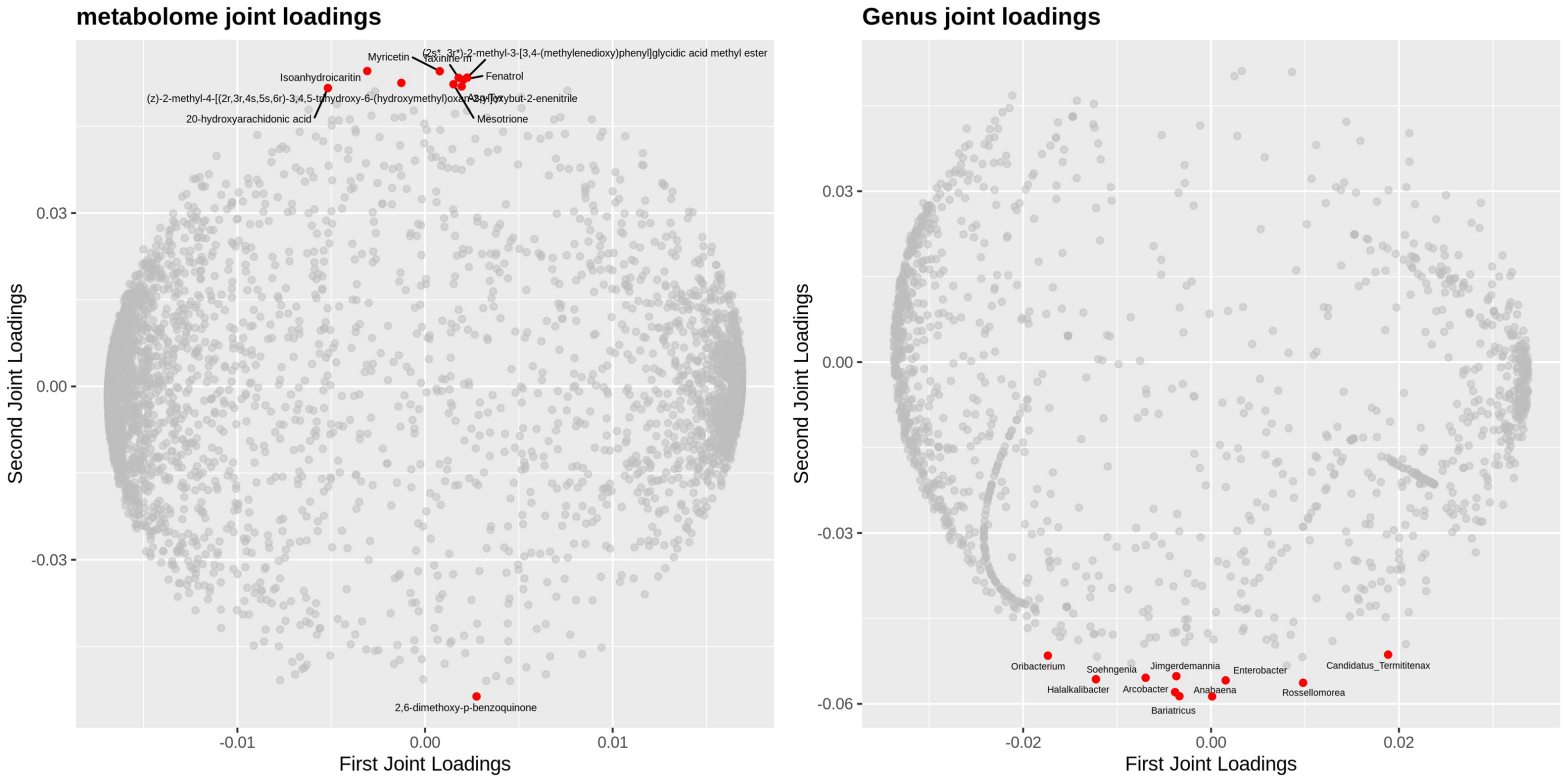
O_2_PLS loading plot of two omics datasets at the genus level.

O2PLS (Orthogonal Partial Least Squares), an extension of the orthogonalized partial least squares framework, enables joint modeling and interaction prediction for dual-omics data matrices. This analytical approach integrates multidimensional metagenomic and metabolomic datasets to effectively uncover latent associative mechanisms between host gut microbial communities and metabolomic profiles. It not only evaluates the global correlation strength between two omics datasets but also precisely identifies core microbial taxa or metabolic biomarkers driving these associations. In the preprocessing phase, rare taxa with relative abundances below 0.1% were first filtered out. Subsequently, using microbial abundance data across all taxonomic levels (phylum to species) and metabolite concentration matrices, O2PLS-based multidimensional statistical modeling was performed via the R-based OmicsPLS package to systematically dissect microbe-metabolite interaction networks. Loading plots for both omics datasets (Figures 16, 17) were generated to visualize feature importance. Results demonstrated that at the phylum level, Spirochaetota, Myxococcota, and Fibrobacterota exhibited high loading values in the joint analysis, indicating their significant contributions to sample stratification. Notably, Candidatus_Aenigmarchaeota and Candidatus_Parcubacteria showed elevated scores in the second principal component. Among metabolites, Asp-Tyr and omega-hydroxyemodin displayed strongly positive loadings in the first principal component, whereas thiosulfuric acid and *α*-guanidinoglutaric acid were associated with significant negative loadings.

At the genus level, the top 10 microbial genera most strongly associated with metabolomics exhibited significant negative loadings in the second principal component. Among these, CandidatusTermitienax and Rossellomorea demonstrated strongly positive loadings in the first principal component, whereas Oribacterium, Halalkalibacter, Soehngenia, and Arcobacter displayed pronounced negative loadings. Among metabolites, all top 10 metabolites except 2,6-dimethoxy-p-benzoquinone showed a marked positive loading trend in the second principal component. Compounds including 2,6-dimethoxy-p-benzoquinone, 20-hydroxyarachidonic acid, isoanhydroicaritin, myricetin, fenatrol, taxinine, and mesotrione exhibited high loading values in the joint loading analysis.

#### 3.5.2 Pearson correlation coefficient analysis

**Figure 18:**
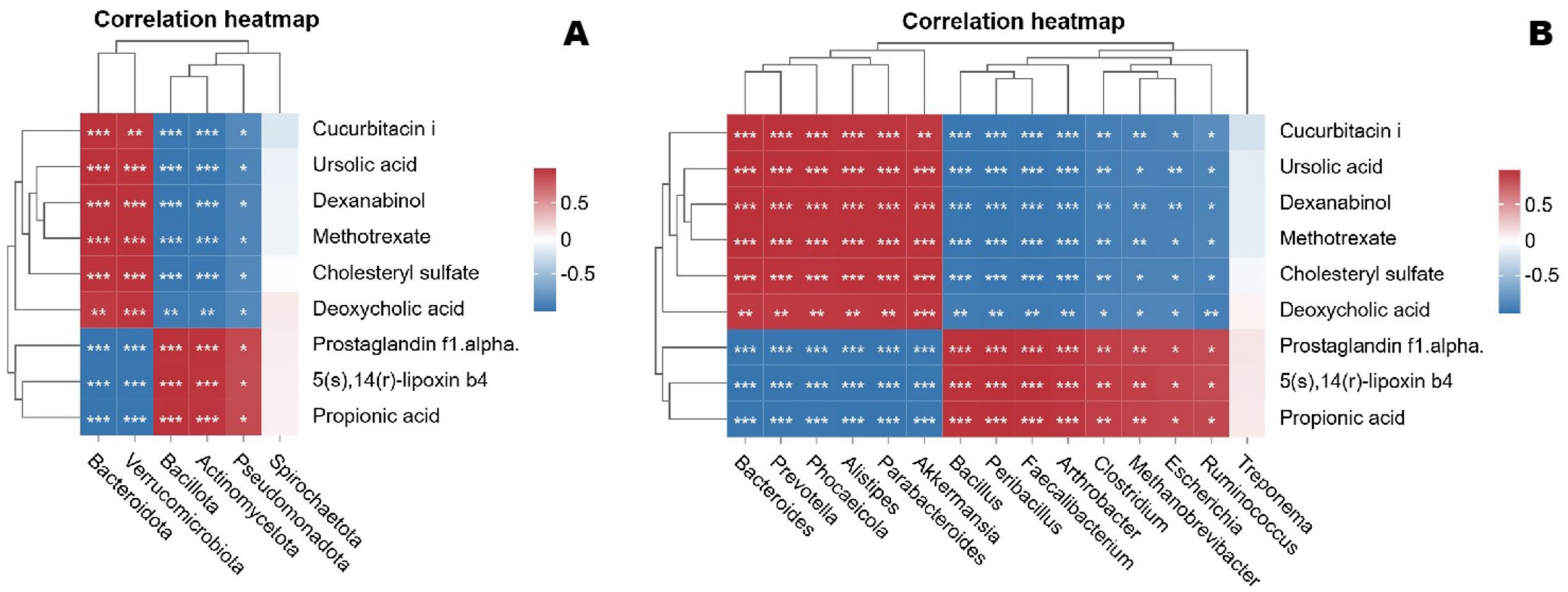
Correlation heatmap of two omics datasets at the phylum **(A)** and genus **(B)** levels.

Based on the O2PLS model analysis results, we employed the Pearson correlation coefficient to quantify the linear association strength between the two variable groups. This statistical measure reflects covariation trends between variables through the ratio of covariance to standard deviation, with values ranging from [−1, +1], where the absolute value correlates positively with association strength. In this study, we applied this analytical approach to assess associations between gut microbial abundances across taxonomic levels (phylum to species) and metabolite concentrations in Tibetan sheep, focusing on deciphering microbiome-metabolome interaction patterns under seasonal grouping conditions (cold/warm seasons). During implementation, bivariate correlation analysis was performed using the cor.test function in R, followed by Fisher’s Z-transformation of raw correlation coefficients to stabilize variance. Final significance (*p*-values) was calculated based on transformed statistics to control false positive errors under multiple testing conditions. Building on preliminary screening of microbial and metabolite abundances and functions, key phyla, genera, and metabolites were selected for detailed analysis (Figure 18). At the phylum level, Bacteroidota and Verrucomicrobiota showed significant positive correlations with cucurbitacin I, ursolic acid, dexanabinol, methotrexate, cholesteryl sulfate, and deoxycholic acid, while exhibiting negative correlations with prostaglandin F1α, 5(S),14(R)-lipoxin B4, and propionic acid. Conversely, Bacillota, Actinomycetota, Pseudomonadota, and Spirochaetota displayed inverse correlation trends with these metabolites.

At the genus level, *Bacteroides*, *Prevotella*, *Phocaeicola*, *Alistipes*, *Parabacteroides*, and *Akkermansia* showed significant positive correlations with metabolites including Cucurbitacin I, Ursolic acid, Deshanols, Methotrexate, Cholesteryl sulfate, and Deoxycholic acid, while displaying significant negative correlations with Prostaglandin F1α, 5(S),14(R)-lipoxin B4, and Propionic acid. Conversely, other genera such as *Bacillus*, *Arthrobacter*, *Clostridium*, *Treponema*, *Methanobrevibacter*, *Peribacillus*, *Escherichia*, *Ruminococcus*, and *Faecalibacterium* exhibited opposite correlation trends with these metabolites.

## 4 Discussion

### 4.1 Effect of climatic changes during cold and warm seasons on serum biochemical indices in Tibetan sheep

Alanine aminotransferase (ALT), a core enzyme for amino acid metabolism in animals, is primarily localized in hepatocytes (12). Warm-season Tibetan sheep showed significantly higher serum ALT concentrations than cold-season counterparts, likely due to increased protein intake from abundant forage enhancing hepatic amino acid metabolism and ALT activity for amino group transfer. Total cholesterol (TC), a critical cell membrane component and bile acid precursor, is ~70–80% synthesized in the liver (13). Elevated warm-season TC suggests enhanced lipid biosynthesis for energy storage ahead of cold-season shortages. Creatinine (CRE), the metabolic end product of creatine/phosphocreatine linked to muscle energy reserve (14), is inversely correlated with glomerular filtration rate (GFR) (15). Higher warm-season CRE may result from increased muscle activity/energy metabolism promoting creatine breakdown and/or elevated water intake. Blood urea nitrogen (BUN), a protein metabolism end product influenced by intake/synthesis/excretion (16), was higher in warm-season sheep due to increased dietary protein-driven urea production via the urea cycle and heat-induced dehydration reducing renal excretion efficiency.

### 4.2 Effects of seasonal phenological changes on gut microbiota composition and function in Tibetan sheep

Significant differences in the community structures of bacteria, archaea, and fungi were observed in the gut microbiota of Tibetan sheep between cold and warm seasons. At the bacterial phylum level, Bacteroidota, Bacillota, Actinobacteriota, and Verrucomicrobiota dominated the intestinal ecosystem, indicating their critical functional roles. While gut microbiota composition remains stable within hosts due to genetic factors, relative abundances adapt to environmental changes (17–19). Bacteroidota and Verrucomicrobiota showed significantly higher relative abundances in warm-season sheep. Bacteroidota, a carbohydrate-degrading phylum, secretes cellulases and hemicellulases to break down plant polysaccharides into absorbable monosaccharides or short-chain fatty acids (SCFAs), enhancing nutrient utilization and host immunity (20) Their increased abundance in the warm season likely reflects abundant forage with high cellulose content. Verrucomicrobiota, represented by *Akkermansia*, degrades mucin in the intestinal mucus layer and ferments polysaccharides to produce SCFAs while modulating gut immunity (21, 22), with elevated abundances linked to warm-season polysaccharide-rich diets. Conversely, Bacillota and Actinobacteriota were significantly more abundant in cold-season sheep (*p* < 0.05). Bacillota, Gram-positive spore-forming bacteria, degrade cellulose/hemicellulose and ferment carbohydrates into SCFAs for energy provision (23). Actinobacteriota, with branched mycelial structures, maintain metabolic activity under low temperatures and degrade cellulose/lignin to enhance fiber utilization while producing antibiotics and bioactive compounds to suppress pathogens and regulate immunity (24, 25). The cold-season enrichment of Bacillota and Actinobacteriota likely results from their environmental adaptability and organic matter degradation capabilities to sustain nutritional metabolism under harsh conditions, with *Arthrobacter* and other *Actinobacteriota* genera further modulating intestinal permeability and immunity via antibiotic production to reduce disease risks (26).

On a broader ecological perspective, these shifts indicate that Tibetan sheep leverage their gut microbiota to maximize nutrient extraction and maintain health under seasonal stress. Similar strategies have been observed in other high-altitude ruminants such as yaks, wherein Firmicutes and Bacteroidetes consistently dominate the gut community to support fiber digestion (27). However, differences in seasonal microbiota responses between species are evident. For instance, Tibetan sheep in this study exhibited higher relative abundance of Bacteroidota during the lush warm season, whereas yaks have been reported to show increased Bacteroides (a key Bacteroidota genus) during harsh winters with limited forage. However, differences in seasonal microbiota responses between species are evident. For instance, Tibetan sheep in this study exhibited higher relative abundance of Bacteroidota during the lush warm season, whereas yaks have been reported to show increased Bacteroides (a key Bacteroidota genus) during harsh winters with limited forage (28). Such disparities may reflect species-specific feeding behaviors and metabolic needs – Tibetan sheep possibly emphasize rapid energy storage from abundant summer forage, while yaks rely on robust fiber-degrading communities to survive winter scarcity. Nonetheless, both animals share a reliance on fiber-fermenting taxa as a possible adaptive association with high-altitude survival, highlighting the potential role of Bacteroidota and Firmicutes in cellulose breakdown and SCFA production. Furthermore, the elevated warm-season Verrucomicrobiota (i.e., Akkermansia) in Tibetan sheep suggests enhanced mucus utilization and gut barrier maintenance when nutrient intake is high. Akkermansia is recognized as a beneficial microbe that strengthens intestinal barrier function and modulates host immunity, potentially contributing to improved nutrient absorption and health during the summer growth phase. By contrast, the winter-dominant Bacillota (Firmicutes) likely include many butyrate-producing bacteria (e.g., Ruminococcaceae spp.), which yield SCFAs like butyrate that fuel colonocytes and exhibit anti-inflammatory effects, thereby preserving gut integrity under nutritional stress. These observations suggest that the observed compositional changes are not merely taxonomic oscillations but may also reflect functionally meaningful adaptations, aligning with known trends in plateau ruminants while also emphasizing unique Tibetan sheep-specific microbiota dynamics.

Significant phylum-level compositional similarities and seasonal differences were observed in the gut archaeal communities of Tibetan sheep between cold and warm seasons (29). Euryarchaeota dominated both seasons, known for methane production and hydrogen metabolism regulation to sustain cellulose degradation and SCFAs production while balancing microbial communities and maintaining intestinal anaerobiosis (30). Its core genus *Methanobrevibacter* also demonstrated trans-seasonal dominance, highlighting its irreplaceable role in hydrogen metabolism and microecological balance. Thermoproteota abundance was significantly higher in warm-season sheep (*p* < 0.05), with active *Thermosphaera* and *Thermococcus* likely linked to enhanced forage fermentation and thermoadaptive metabolic demands (31). The subdominance of *Methanosphaera* in cold seasons may indicate methylotrophic methane production adaptation to limited hydrogen availability under low temperatures. Concurrently, this study revealed high synergy between seasonal adaptation and functional niches in gut fungal communities. Ascomycota, the core functional phylum, maintained trans-seasonal dominance likely due to its lignocellulolytic enzyme (laccase, cellulase) secretion capabilities critical for plant cell wall degradation (32). Its abundance advantage during warm seasons with elevated forage fiber content may enhance host hemicellulose utilization efficiency for energy gain.

Functional annotation analysis based on egg NOG and KEGG revealed dual metabolic characteristics of “intestinal homeostasis maintenance” and “seasonal strategy differentiation” in Tibetan sheep gut microbiota between cold and warm seasons. Egg NOG analysis showed high conservation in fundamental functions such as replication, recombination and repair, and carbohydrate transport/metabolism (23 pathways with similar abundances), indicating that stable core metabolic modules underpin gut microecological balance. However, differential KEGG level 3 pathways (6 warm-season-specific vs. 31 cold-season-specific) uncovered adaptive metabolic mechanisms to phenological stressors. Warm-season enrichment of Carbon fixation pathways in prokaryotes likely converts CO₂ to organic matter via the Calvin-Benson-Bassham (CBB) cycle (33) or reductive tricarboxylic acid cycle (34), potentially fueled by photosynthate inputs (e.g., organic acids) from lush forage to supplement microbial carbon sources. Upregulated Other glycan degradation pathways suggested enhanced capacity to hydrolyze non-cellulosic plant cell wall polysaccharides (e.g., pectin, xylan), abundant in fresh warm-season forage, thereby releasing fermentable substrates to synergize with host energy demands (35). Cold-season enrichment of Carbon metabolism and Pyruvate metabolism reflected adaptation to high-fiber diets (36), with activated glycolysis and pentose phosphate pathways prioritizing ATP/reducing power generation from limited carbon sources to offset energy loss under low temperatures (37). Enhanced ABC transporters indicated upregulated transmembrane systems (e.g., oligosaccharide/amino acid transporters) to improve nutrient scavenging efficiency amid reduced forage nutritional diversity (38). Elevated Methane metabolism pathways functionally aligned with increased Euryarchaeota (e.g., Methanobrevibacter) abundances, where hydrogen-consuming methanogenesis maintained low hydrogen partial pressure to optimize cellulolytic bacterial activity, forming cross-domain metabolic coupling (39).

Significant activation of DNA replication and mismatch repair pathways in cold-season gut microbiota may reflect increased DNA damage risks under low temperatures (e.g., oxidative stress or replication errors), with microbes enhancing genetic fidelity to maintain genomic stability (40). Upregulated aminoacyl-tRNA biosynthesis likely supports rapid synthesis of cold-shock proteins via translational optimization (41). Warm-season microbiota maximize high-quality forage utilization through synergistic Carbon fixation and Other glycan degradation to meet host growth/reproduction demands, while cold-season communities sustain energy homeostasis under resource limitation via expanded Carbon metabolism networks and stress adaptation mechanisms (e.g., Methane metabolism, DNA repair). This seasonal metabolic strategy switch may represent a possible microbiological association with Tibetan sheep adaptation to plateau extremes. Differentiated metabolic pathways suggest that gut microbiota may be involved in host energy acquisition/allocation strategies through dynamic functional module reorganization, rather than merely passively responding to environmental changes.

### 4.3 Effects of climatic changes during cold and warm seasons on the metabolite composition and function of intestinal flora in Tibetan sheep

The dynamic divergence of gut microbiota metabolites in Tibetan sheep between cold and warm seasons reflects adaptive adjustments in host-microbe co-metabolic strategies during phenological shifts. At the nutrient metabolism level, cold-season metabolite profiles are characterized by significant enrichment of SCFAs and propanoate metabolism pathways. SCFA production relies on anaerobic fermentation of plant fibers by gut microbiota (42), with elevated neutral detergent fiber (NDF) and acid detergent fiber (ADF) content in cold-season forage providing abundant fermentation substrates. SCFAs not only serve as the primary energy source for intestinal epithelial cells but also regulate host metabolism through multiple pathways, such as activating G protein-coupled receptors 41/43 (GPR41/43) to promote expression of tight junction proteins, enhancing intestinal permeability and barrier integrity (43). SCFAs also participate in glucose metabolism and modulate adipocyte differentiation and lipid storage gene expression (44, 45) The increased SCFA content in cold-season intestinal metabolites suggests Tibetan sheep prioritize energy allocation for thermoregulation over adipose tissue deposition during cold periods. Concurrently, cold-season upregulation of stearoylcarnitine, a long-chain fatty acid transport carrier, further indicates microbial enhancement of mitochondrial *β*-oxidation to address high energy demands in low-temperature environments (46). In contrast, warm-season metabolic networks focus on biosynthesis of secondary bile acids (e.g., deoxycholic acid) and unsaturated fatty acids (e.g., oleic acid). Studies show that secondary bile acids inhibit hepatic cholesterol synthesis by activating the farnesoid X receptor (FXR) and stimulate intestinal epithelial proliferation via the G protein-coupled bile acid receptor 5 (TGR5), optimizing host lipid digestion and absorption efficiency (47, 48). Additionally, unsaturated fatty acids promote adipocyte differentiation and lipid deposition through peroxisome proliferator-activated receptor gamma (PPARγ) (49), aligning with energy storage strategies during resource-rich warm seasons. The significant enrichment of steroid hormone biosynthesis pathways may enhance calcium and phosphorus absorption and skeletal development by regulating fat-soluble vitamin metabolism (e.g., vitamin D3), thereby supporting host reproductive and growth demands (50).

At the level of immune regulation, the significant enrichment of prostaglandin E2/F1α in the cold-season gut may arise from its dual roles: on one hand, suppressing Th1 cell differentiation while promoting regulatory T lymphocyte (Treg) proliferation to alleviate cold stress-induced mucosal inflammation (51); on the other hand, prostaglandin E2 likely inhibits neutrophil infiltration by activating the prostaglandin F receptor (FP) (52), mitigating intestinal oxidative damage caused by cold stress under plateau hypoxia. Concurrently, melatonin accumulation maintains oxidative stress homeostasis through free radical scavenging and NF-κB pathway inhibition (53, 54). In contrast, warm-season upregulation of plant secondary metabolites such as luteolin-7-glucoside and ursolic acid not only suppresses pathogen proliferation via direct antimicrobial effects (55) but also enhances host antioxidant defenses through the Nrf2 pathway (56), countering ecological pressures from heightened pathogen activity. Notably, seasonal differences in proline-tryptophan (cold season) versus Pro-Pro/Pro-Thr (warm season) reveal microbiota-mediated immunomodulatory mechanisms involving tryptophan metabolism and dipeptide signaling. The former may influence intestinal immune tolerance via the kynurenine pathway (57), while the latter potentially modulates innate immune response thresholds as TLR pathway ligands (58).

Comparatively, previous research on yaks has reported somewhat different seasonal metabolite patterns, underscoring the unique metabolic strategy of Tibetan sheep. For example, free-grazing yaks exhibit peak total SCFA concentrations during the nutrient-rich summer, which correlates with heightened immune responses (28). In contrast, our results show that Tibetan sheep accumulate more SCFAs during winter, suggesting a greater reliance on microbial fermentation for energy during cold stress. This discrepancy might be attributed to species-specific ecology and physiology: yaks, being larger, can store substantial energy reserves and thus synchronize peak SCFA production with summer immunity boost, whereas the smaller Tibetan sheep must continuously ferment fibrous forage to meet immediate thermogenic needs in winter. Nonetheless, SCFAs emerge as a common key metabolite for high-altitude adaptation in both species – notably, Tibetan sheep and other plateau herbivores produce higher SCFA levels than their lowland counterparts (28). These SCFAs not only provide critical energy but also signal through receptors like GPR41/GPR43 to strengthen the gut barrier and induce anti-inflammatory pathways, which is crucial for maintaining homeostasis under extreme conditions. Similarly, secondary bile acids appear to play beneficial roles across different ruminants; by facilitating lipid digestion and regulating host metabolism (e.g., via FXR and TGR5 signaling) (27), the enhanced bile acid biosynthesis in warm-season Tibetan sheep likely parallels adaptive processes in yaks as they prepare for winter fat storage. Another noteworthy metabolite is melatonin – a potent antioxidant and immunomodulator. Interestingly, yak studies documented higher systemic melatonin levels in summer (coinciding with abundant nutrition and elevated immune activity), whereas Tibetan sheep in our study showed a gut melatonin surge in winter. Given melatonin’s known free-radical scavenging and anti-inflammatory effects (59), its winter elevation in Tibetan sheep could be a tailored response to mitigate cold-induced oxidative stress. This contrast with yaks may stem from differences in photoperiod sensitivity or the extent of microbial melatonin production in the gut. Taken together, these comparisons highlight that while the broad strategy of leveraging microbial metabolites for energy and immune balance is conserved among plateau ruminants, the timing and magnitude of specific metabolite shifts are species-specific. Our study thus underscores Tibetan sheep’s distinctive metabolic adjustments – particularly the winter-focused SCFA and melatonin boost – as an evolutionary fine-tuning to the Qinghai-Tibetan Plateau’s challenges.

### 4.4 Combined analysis of macrogenomics and untargeted metabolomics

The O2PLS model results indicate that the core taxonomic units of Tibetan sheep gut microbiota at the phylum level are Spirochaetota and Fibrobacterota, suggesting their critical roles in energy acquisition and nutrient metabolism. The hydrogenotrophic metabolism of Spirochaetota and its synergistic relationship with methanogens (e.g., Methanobrevibacter) may enhance energy harvesting, as cellulose-derived hydrogen production under anaerobic fermentation has been documented (60). Concurrently, Fibrobacterota, as specialized cellulose-degrading anaerobes (61), upregulate cellulase-specific gene expression to enhance intestinal fiber digestion efficiency. In metabolomic data, the high loading values of Asp-Tyr and omega-hydroxyemodin may arise from their ability to activate the mTORC1 signaling pathway to promote intestinal epithelial proliferation (62) or stimulate the Nrf2 pathway (63) to bolster oxidative stress resistance in high-altitude environments. Thiosulfate, which can be reduced to hydrogen sulfide (64) or serve as a metabolic substrate for sulfate-reducing and sulfur-oxidizing bacteria (65), regulates intestinal sulfur cycling, enhances fiber degradation, protects epithelial cells from oxidative damage, and modulates inflammatory responses to reinforce mucosal barrier function (66). Its negative loading in this study may reflect its role as a sulfur metabolic intermediate in repairing mucosal barriers or fine-tuning immune homeostasis. At the genus level, the positive loadings of *Candidatus Termitienax* and *Rossellomorea* suggest their potential involvement in high-altitude immune regulation via terpenoid biosynthesis (e.g., taxinine) (67).

Members of Bacteroidota secrete multiple cellulases to efficiently degrade forage cellulose, hemicellulose, and pectin into short-chain fatty acids (SCFAs) (68). They also produce propionic acid via fermentation, which has been reported to be associated with GPR41-related signaling, inhibition of the NF-κB pathway, reduced pro-inflammatory cytokine release, and modulation of host immunity (69). Additionally, Bacteroidota decompose plant proteins into amino acids (70), supporting intestinal mucosal repair and immune cell proliferation (71). Verrucomicrobiota promote intestinal mucus layer metabolism and barrier maintenance (72), regulate host immune responses and inflammation (73), and participate in nutrient metabolism (74). Pearson correlation network analysis in this study suggested that Bacteroidota and Verrucomicrobiota were positively associated with secondary bile acid biosynthesis, which may be related to enhanced nutrient metabolism in the warm season. Their significant negative correlation with propionic acid likely reflects adaptive metabolic shifts: cold-season reliance on SCFA functions vs. warm-season lipid storage. Positive correlations between Bacillota and Prostaglandin F1α may involve collaborative intestinal mucosal barrier repair during cold stress. Bacillota produce butyric acid to activate host prostaglandin synthesis pathways (75), promoting Prostaglandin F1α production. This metabolite activates endothelial cell prostaglandin receptors (76), enhancing mucosal microcirculation to supply oxygen/nutrients to damaged areas while inhibiting the NF-κB pathway to reduce inflammatory mucosal damage. Significant positive correlations between Actinobacteriota and Methotrexate suggest modulation of folate metabolism cycles affecting host hematopoiesis (77). Actinobacteriota genomes contain complete folate biosynthesis pathways, with metabolites like 5-methyltetrahydrofolic acid (5-MTHF) acting as competitive antagonists of Methotrexate by inhibiting dihydrofolate reductase (DHFR) activity to regulate erythropoietin (EPO) signaling (78). At the genus level, *Bacteroides* and *Akkermansia* displayed intricate metabolic cooperation. *Bacteroides* secrete *β*-xylosidase to degrade forage xylan into xylo-oligosaccharides (XOS) (79), a critical substrate for *Akkermansia* to degrade mucopolysaccharide protein complexes. Conversely, *Akkermansia*-produced acetate inhibits Bacteroides sulfatase activity (3), forming a negative feedback loop. This synergistic-antagonistic interaction maintains intestinal homeostasis by promoting mucus layer regeneration and inhibiting bile acid production.

### 4.5 Limitations and future directions

Although our multi-omics analyses provide valuable insights into seasonal shifts in the gut ecosystem and host metabolic adaptation in Tibetan sheep, several limitations should be acknowledged. First, samples were collected from a single geographic region and at only two seasonal time points (cold vs. warm season), which may not capture interannual variability or broader environmental heterogeneity. Second, despite the relatively robust associations observed between key differential taxa (e.g., Bacteroidota, Bacillota, Akkermansia) and core metabolites (e.g., SCFAs, secondary bile acids, melatonin), the observational nature of this study precludes causal inference. Future research should therefore incorporate longitudinal sampling across multiple years and regions, extend sampling to multiple gastrointestinal compartments, and integrate precise dietary intake measurements with detailed environmental metadata to improve generalizability and interpretability. Importantly, mechanistic validation is needed to establish the “microbiota–metabolite–host phenotype” causal chain, for example by applying targeted metabolomics with absolute quantification of SCFAs and bile acids, combining microbial isolation/cultivation with gnotobiotic or microbiota-transfer experiments, and conducting controlled intervention trials (e.g., supplementation strategies that enhance SCFA production or modulate bile acid metabolism). These efforts will help translate our findings into actionable approaches for optimizing cold-season nutritional management and husbandry of Tibetan sheep.

## 5 Conclusion

In this study, we examined Tibetan sheep on the Qinghai–Tibet Plateau across cold and warm seasons and identified significant seasonal differences in serum biochemical indices, gut microbiota composition, and associated metabolites. The variations in serum biochemical parameters likely reflect adaptive responses to seasonal phenological changes. Structural and functional shifts in the gut microbiota indicate a transition in nutritional strategy from “nutrient maximization” during warm seasons to “survival prioritization” in cold seasons. Differences in microbial metabolite profiles and related functions may stem from metabolites that drive host physiological remodeling through modulation of the energy-immunity axis. Ultimately, gut microbiota and their metabolites in Tibetan sheep work in concert to sustain host energy supply and immune regulation under extreme plateau environmental conditions.

## Data Availability

The original contributions presented in the study are publicly available. These data can be found in repository: OMIX database of NGDC. The OMIX accession number is: OMIX009476.
